# Barriers and facilitators to implementation of nutrition-related actions in school settings in low- and middle-income countries (LMICs): a qualitative systematic review using the Consolidated Framework for Implementation Research (CFIR)

**DOI:** 10.1186/s43058-023-00454-y

**Published:** 2023-06-27

**Authors:** Biljana Meshkovska, Mekdes Kebede Gebremariam, Prudence Atukunda, Per Ole Iversen, Margareta Wandel, Nanna Lien

**Affiliations:** 1 Department of Nutrition, Institute of Basic Medical Sciences, Faculty of Medicine, University of Oslo, Blindern, P.O. Box 1046, 0316 Oslo, Norway; 2 Department of Community Medicine and Global Health, Institute of Health and Society, University of Oslo, Kirkeveien 166, Fredrik Holsts hus, 0450 Oslo, Norway; 3 Center for Crisis Psychology, University of Bergen, Møllendalsbakken 9, 5009 Bergen, Norway; 4 Department of Haematology, Oslo University Hospital, Sognsvannsveien 20, 0372 Oslo, Norway; 5 Division of Human Nutrition, Stellenbosch University, Francie Van Zijl Drive, Tygerberg, Cape Town, South Africa

**Keywords:** Barrier, Facilitator, Implementation, Double-duty, Nutrition, Action, School, LMIC, CFIR

## Abstract

**Background:**

Low- and middle-income countries (LMICs) are particularly vulnerable to the double burden of malnutrition: co-existence of underweight, overweight, obesity, and/or diet-related non-communicable diseases. Nutrition-related double-duty actions in school settings have been identified as one of the ways to address this challenge. However, to be able to take full advantage of the potential impact, it is important to understand their implementation as well. The aim of this paper is to systematically review qualitative research on barriers and facilitators to the implementation of nutrition-related actions in the school settings in LMICs.

**Methods:**

The following databases were searched: EMBASE, ERIC, MEDLINE, Global Health and PsycInfo (all on Ovid), Scopus (Elsevier), the Web of Science Social Sciences Citation Index, and Global Index Medicus from the World Health Organization. Of the 4253 identified records, 4030 were excluded after the abstract and title screen, leaving 223 for the full-text screen. A final 36 papers were included in this review. The consolidated framework for implementation research (CFIR) was used in the analysis.

**Results:**

We identified barriers and facilitators to implementation linked to the following CFIR constructs/sub-constructs: design quality and packaging, cost (intervention characteristics); target group needs and resources, cosmopolitanism, external policy and incentives (outer setting); structural characteristics, readiness for implementation (inner setting); knowledge and beliefs (characteristics of individuals) and engaging, executing (process). All identified constructs apart from target group needs and resources, knowledge and beliefs, and engaging were predominantly barriers. Available resources were the most prevalent barriers across studies.

**Conclusion:**

This review identified barriers and facilitators to the implementation of nutrition-related actions based on qualitative articles in the school setting in LMICs, using the CFIR. Schools face continuous challenges in regard to funding and the government sector may have a role to play not only by offering financial assistance, but also through policy-making that would support healthy eating practices on school grounds.

**Registration:**

PROSPERO ID: CRD42022296159.

**Supplementary Information:**

The online version contains supplementary material available at 10.1186/s43058-023-00454-y.

Contributions to the literature

- Awareness of the needs of children and families facilitates the implementation of nutrition-related actions in school settings in LMICs.
- Belief in the positive influence of nutrition-related actions in schools, by teachers, principals, and children and parents, facilitates the implementation of such actions.
- Schools lack resources, and this is one of the most prevalent barriers to the implementation of nutrition-related actions in schools in LMICs.
- Overcoming barriers to the implementation of nutrition-related actions in school settings in LMICs requires the cooperation of different actors: from the teachers, principals, children, and parents at the schools to community leaders and the government sector.

## Background

The double burden of malnutrition is understood as the co-existence of underweight along with overweight, obesity, or diet-related non-communicable diseases (NCDs) at individual, household, and population levels and across the lifespan [1]. Low- and middle-income countries (LMICs) are particularly vulnerable due to the persistence of undernutrition, while undergoing the process of nutrition transition characterized by a diet high in saturated fats, added sugar, and refined foods and thus increased risk of overweight/obesity [2–4]. To address the double burden of malnutrition, the World Health Organization (WHO) has identified double-duty actions as any interventions, programs, and policies which have the potential to simultaneously reduce the risk of undernutrition, as well as overweight, obesity, and diet-related NCDs [5–8].

School-based food policies and programs are one type of double-duty actions which are relevant candidates to address the double burden of malnutrition [2, 5]. Although double-duty actions can be introduced with the explicit aim of addressing the double burden of malnutrition, they do not necessarily have to be new [5]. It is with this in mind that for the purpose of this research, we consider school-based nutrition-related interventions/policies/programs as nutrition-related double-duty actions. Research has found that school-based food policies and programs address around 40% of the drivers of the double burden of malnutrition [2]. To date, there have been reviews aiming at identifying actions targeting the double burden of malnutrition more generally [9] and the effectiveness of preventive school-based obesity interventions in LMICs [10]. There are also published protocols of upcoming reviews which will focus on the effect of school-based interventions on nutritional, educational, and health outcomes of school-age children and adolescents in LMICs [11, 12].

However, to fight the double burden of malnutrition, it is important to understand not only which types of school-based, nutrition-related interventions/policies/programs are effective, but also what may be the best way to implement them. Research has shown that implementation can directly impact the outcome of an intervention/policy/program [13]. Yet, research focusing on such implementation has been scarce. A review by Klinberg and colleagues [14] which looks at the effect and implementation of childhood obesity preventive interventions in Africa found only one process evaluation that provides information on barriers and facilitators to implementation [15]. Another review by Ezezika and colleagues [16] did focus on implementation specifically but primarily provided information on barriers and facilitators to large-scale nutrition interventions in Africa, such as micronutrient powders and community fortification programs. It again identified only a single study focused on implementation in the school setting [15]. Finally, a scoping review by McIsaac and colleagues provides a rich overview of factors influencing the implementation of nutrition policies in schools, however, with an international rather than LMIC focus [17].

With this in mind, the aim of this paper is to systematically review research on barriers and facilitators that influence the implementation of nutrition-related interventions/policies/programs in the school setting in LMICs. The focus will be specifically on qualitative research, since this was found most appropriate for exploring and understating barriers and facilitators in depth. For this purpose, we will use the Consolidated Framework for Implementation Research (CFIR) [18, 19].

## Methods

### Conceptual framework

The CFIR is a determinant framework well suited for the study of barriers and facilitators to implementation [18]. The framework was built on existing theories, and although it is one-dimensional without specifying the relationships between constructs and distinguishing between different ecological levels [18], it captures and clearly defines a comprehensive list of barriers and facilitators [18, 19]. The CFIR is also the most widely used determinant framework in implementation science, which enables the comparison of findings within and across disciplines and in different contexts and settings [20]. Its scope, clarity of constructs, wide usage, and our previous experience with the framework were the main reasons for choosing it for this review. The CFIR consists of 26 constructs across 5 domains: (1) intervention characteristics (intervention source, evidence strength and quality, relative advantage, adaptability, trialability, complexity, design quality and packaging, cost), (2) outer setting (target group needs and resources, cosmopolitanism, peer pressure, external policy and incentives), (3) inner setting (structural characteristics, networks and communications, culture, implementation climate, readiness for implementation), (4) characteristics of individuals (knowledge and beliefs about the intervention, self-efficacy, individual stage of change, individual identification with the organization, other personal attributes), and (5) process (planning, engaging, executing, reflecting, and evaluating) [18, 19]. The CFIR was used for the coding and analysis of the data extracted from the included articles.

### Search strategy

The protocol for this review was registered in PROSPERO (ID: CRD42022296159). Interventions, programs, and policies which were related to nutrition and implemented in school settings in LMICs were included. The target group of the interventions, programs, and policies (from this point forward termed actions) was children from 6 months to 18 years.

The following databases covering health, psychology, education, and interdisciplinary subjects were searched: EMBASE, ERIC, MEDLINE, Global Health and PsycInfo (all on Ovid), Scopus (Elsevier), the Web of Science Social Sciences Citation Index, and Global Index Medicus from World Health Organization. The search consisted of several synonyms for interventions on nutrition, in combination with synonyms for school-related topics and implementation. To identify studies in LMICs, the LMIC filter from Cochrane was used, except in Global Index Medicus where a simpler search strategy was chosen due to search functionality. We searched for both database-specific subject headings and in the fields for title, abstract, and author keywords. Database searches were performed in October 2021. The searches were not limited by language, date, or study design. The search was conducted with the help of an academic librarian (M.Ø.) (see Additional file 1 for search strategy).

### Study selection

The screening process was conducted in two stages, based on the inclusion and exclusion criteria presented in Table 1. The first stage was a title and abstract screen, with all titles and abstracts screened by two authors (B.M. screened all; P.A., M.G., and P.O.I. were second reviewers, while N.L. resolved all conflicts). In case of doubt about a title or an abstract, or when the abstract was missing, a study was included for a full-text screen. The second stage was a full-text screen with all texts screened by two authors (as for abstract screening). At this stage, articles which were systematic reviews, dissertations, protocols, conference abstracts, not in English, or the full text was not available were excluded.

**Table 1:** The inclusion and exclusion criteria for articles on the implementation of nutrition-related actions in school settings and LMICs

Inclusion	• Nutrition-related double-duty action • Nutrition-related intervention/program/policy • Implementation in the school setting • Qualitative results on barriers and facilitators to implementation • Conducted in LMICs
Exclusion	• No nutrition-related intervention/program/policy • Implementation does not happen in the school setting • Only quantitative results • No barriers and facilitators • Not in LMICs After full-text screen: • Not in English • Systematic review, dissertation, protocol, conference abstract • Full text not available

### Quality appraisal

For the purpose of this review, two checklists specifically designed for quality appraisal of qualitative primary studies were combined and used. The Critical Appraisal Skills Programme (CASP) list which consists of ten questions was used as a basis for the evaluation [21]. However, CASP was supplemented with two additional questions on the use of a theoretical framework, and the use of relevant references linked to the topic, from the “Guidelines for authors and reviewers of qualitative studies” [22]. Thus, all included papers were evaluated on a total of 12 points. Five of the total included articles were evaluated by two authors (B.M. and M.W.) while all remaining were evaluated by one author (B.M.). The quality evaluation of five articles by two authors ensured there was a clear understanding by the authors of all 12 points on which the evaluation was based, as well as consistency in regard to the outcomes of the evaluation process. The two authors shared their individual evaluations of the five articles and discussed the results to reach a consensus for each article on all 12 points of evaluation. The agreed-upon evaluation process was followed for all remaining articles by one author (B.M.).

### Data extraction and synthesis

Data related to the characteristics of the articles and qualitative data related to the barriers and facilitators were extracted. Data extraction for ten articles was completed by two authors (B.M. and P.A., P.O.I., M.W., N.L.) while data for the remaining articles were extracted by one author (B.M.). In regard to the characteristics of the articles, the following information was extracted in an Excel sheet: first author and year of publication, title, aim, action description, geographical focus, data collection methods, sample size, participants, and school type. For the extraction of qualitative data relating to barriers and facilitators to implementation, we followed the principles of qualitative meta-synthesis and thematic synthesis of qualitative research in systematic reviews [23, 24]. Thus, all text from the primary articles which was referring to any identified barriers and facilitators was copy-pasted in its original form in a separate Word document for each article included in this review. Word documents were then uploaded to NVivo for deductive coding based on the CFIR, using the codebook provided on the CFIR website [18, 19]. Coding was done by one author with previous experience of using the CFIR in the context of qualitative systematic reviews (B.M.). Once coding was completed the text under each construct for all papers included in the review was extracted into a matrix following the principles of framework analysis [25] (see Additional file 2). Subsequently, the text under each construct was summarized based on meaning, trying to stay as close to the terminology and interpretation of the original paper as possible, and categorized into barriers and facilitators (see Additional file 3). This review was completed in accordance with the ENTREQ checklist [26] (see Additional file 4).

## Results

### Search results and included articles

**Fig. 1:**
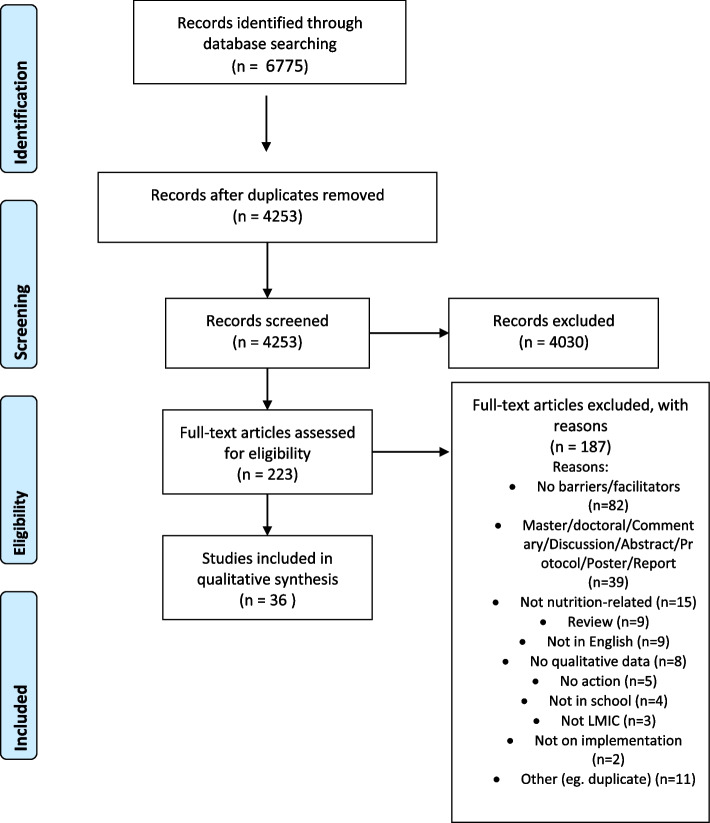
PRISMA flow chart

A total of 6775 records were identified based on the search, with 4253 remaining for title and abstract screen after deduplication (see Fig. 1). Of the 4253 records, 4030 were excluded based on title and abstract screen. Of the 223 remaining for the full-text screen, 187 were excluded with reasons. The most common reason was that no barriers and facilitators were found (*n* = 82). A final 36 articles were included in this review [15, 27–61].

### Quality appraisal

The articles included were of mixed quality. All the included articles had a clear statement of aims, and a qualitative method was considered to be the appropriate choice according to that aim. However, not all discussed why a qualitative method was chosen. Several articles were found to miss key information in regard to the participants such as sample size and recruitment. Very few articles discussed the relationship between researcher(s) and participants, and in eight articles, ethical issues were not mentioned at all. Six articles did not discuss the process of analysis. Finally, 22 articles lacked any reference to a theoretical framework. However, as all articles were evaluated to have a clear statement of findings, and those findings were evaluated to be of value, no articles were excluded based on the quality appraisal. The full quality assessment is found in Additional file 6.

### Article characteristics

An overview of article characteristics is provided in Table 2. The most common type of nutrition-related action described in the included articles was the provision of a school meal (44%), followed by nutrition-related policies coming from the international or national level (28%) and nutrition-related education programs (19%). Studies were most commonly conducted in Brazil (25%), South Africa (17%), and Ghana (14%). Furthermore, in 14 out of the 36 papers included in this review, it was clearly stated that the studied action was targeting children in areas of low socio-economic background. The most common data collection method across papers was interviews (81%), however, often in combination with other methods such as observation (31%), focus group discussions (25%), questionnaire interviews with qualitative data (19%), and document analysis (14%). Finally, the participants of the primary articles were most often school staff (teachers, principals, canteen staff) (78%), followed by persons external to the school setting (such as nutritionists and suppliers) (53%), as well as the children (33%) and parents (22%). Two of the papers examined the preschool setting [41, 46]. For a more detailed description of the characteristics of the articles included see Additional file 5.

**Table 2:** Characteristics of articles included in the review on implementation of nutrition-related actions in school settings and LMICs

	**Number of articles**	**Percentage**
**Type of nutrition action**^a^		
School meal	16	44
Nutrition-related policy (coming from international (e.g., World Health Organization: Health Promoting Schools) or national level (e.g., guidelines for school meals)	10	28
Nutrition-related education program	7	19
Nutrition-related training for school staff	3	8
Environmental action	1	3
Food subsidy	1	3
Tools to implement school meals	1	3
**Country/geographical region**		
Brazil	9	25
South Africa	6	17
Ghana	5	14
India	3	8
Indonesia	2	6
Mexico	2	6
Nepal	2	6
Tanzania	2	6
Cambodia, the Caribbean, China, Costa Rica, Kenya, Malaysia, Marshall Islands, Peru, the Philippines, Samoa, Sri Lanka, Thailand, Uruguay, West Bank and Gaza	1 (per country)	3
**Data collection method**		
Interviews	29	81
Observation	11	31
Focus group discussions	9	25
Questionnaire (with open-ended questions which offered qualitative data)	7	19
Document analysis	5	14
Not clear	1	3
**Participants of the primary article**		
School staff (e.g., teachers, principals, canteen staff)	28	78
External staff (e.g., nutritionists, suppliers)	19	53
Children	12	33
Parents	8	22

^a^One paper can contain more than one type of nutrition action, geographical focus, data collection method, and participants of the primary study

### Barriers and facilitators

**Fig. 2:**
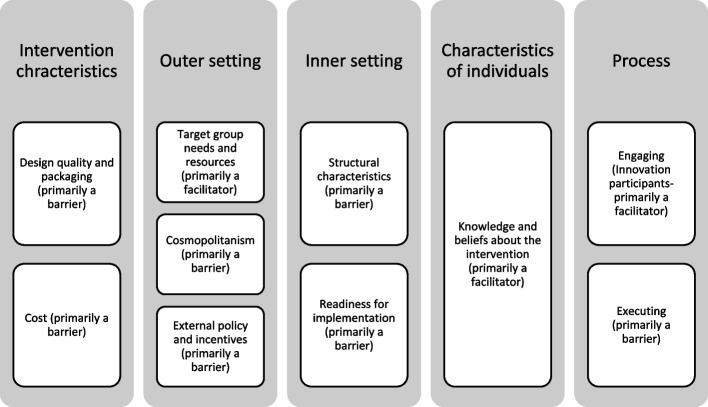
Overview of main findings [18]

Figure 2 provides a visual overview of the main findings across the five domains of the CFIR. We included those constructs/subconstructs which were found to be present in at least 1/3 of the included articles (12 out of the 36 papers). The identified barriers and facilitators for each of the constructs/subconstructs are discussed under each CFIR domain (for summary see Table 3).

**Table 3:** Summary of CFIR constructs identified as barriers and facilitators

Domain	Construct	Sub-construct	Specific barriers	Specific facilitators
Scheme characteristics	Design quality and packaging		Number of articles: 15	Number of articles: 10
			• Lack of diversity in products; repetitive menu; a single menu not considering dietary limitations or cultural diversity • No fruit and vegetables provided • Quantity of products and food not sufficient • No quality criteria for products and food served; use of low-quality food; use of agro-chemicals • Taste of healthy food described as sour and appearance not appealing • Food waste • No variety in educational activities	• Use of fresh products • Products of good quality, with good nutritional value • Homemade food • Use of local products • Less chemicals used, only organic fertilizers • Diverse snacks available • Menu-based cooking • Info provided to teachers on health damages from sugar-sweetened beverages and processed food • Use of videos in educational activities • Health education for children
	Cost		Number of articles: 11	Number of articles: 1
			• Healthy food perceived as expensive • Short shelf life of healthy food makes it more expensive • High cost of fruit and vegetables • High cost of homemade products • Higher cost of products coming from family farmers • Funds provided not sufficient for nutritional food; not sufficient to cover all children; not sufficient to cover all components of a nutritional program • No adjustment of funds provided after inflation; economic crises raised prices of food without change in funds provided	• Government and farmers invest in production
Outer setting	Target group needs and resources		Number of articles: 5	Number of articles: 8
			• When food is not provided for free, preference to provide cheap food with low nutrition value as it is what the children can afford • Children from poor households are not familiar with the use of tap water which makes some education interventions difficult • Differences in quality, quantity, and frequency of meals when parents contribute to feeding programs; children most in need either excluded or if food was provided to all independent of parental contribution de-motivation of those parents who contributed • When children live in poverty, it is difficult to implement dietary changes, eat healthy food	• Free school meals satisfy the physiological and nutritional needs of children from poor communities • School meal may be the only meal during the day for some children, especially during economic crises • Having the school meal is a reason some parents send their children to school • Parents do not have to give children snack money, nor lunch at home when they receive it for free at school, thus saving some resources; children buy less unhealthy food as they are not given snack money • In some cases, school meals benefit families as well, as leftovers are sent home with the children • School meals provide income for local family farmers • School meals promote social integration of students • Health and nutrition-related programs in schools give access to such services to children of lower socio-economic background
	Cosmopolitanism		Number of articles: 11	Number of articles: 8
			• Lack of cooperation between principals and cooperatives providing food products to schools, with the cooperatives being perceived as not reliable, not delivering food on time, and not handling the administration of documents well by principals of schools • Lack of cooperation between teachers and caterers • When suppliers deliver food late, conflict between teachers, suppliers, and those preparing the food • Lack of cooperation between schools in regard to the design and implementation of education-related activities • Partnerships between schools, industry, and vendors on school property, where unhealthy food is made available in return for financial support by industry to schools • Lack of involvement of the health sector in schools • Lack of involvement of schools in decisions by the education sector • When nutritionists not present as an external actor, lack of cooperation between principals, parents, and teachers in monitoring feeding programs	• Cooperation between food kiosks and schools in obesity prevention programs • Cooperation between teachers and caterers • Cooperation between the community and schools in implementing school meals; raising funds for nutrition programs from the community • Cooperation between schools and nutritionists in producing menus for school feeding • Cooperation between the community, schools, parents, and health sector • Cooperation between teachers, parents, cooks, and local cooperatives • Cooperation between schools and health and education sectors in providing nutrition guidance • Cooperation between schools and farmers; produce for schools bought from local farmers • Local women prepare food for schools
	External policy and incentives		Number of articles: 12	Number of articles: 5
			• No legal sanctions for non-compliance • Schools and principals finding out about guidelines and new food policies either through a simple notice with no added information or from the news • No guidance offered on the implementation • No incentive to implement without additional funding • Industry lobbying against any policies toward sugar-sweetened beverages • Policies allowing industry to donate to schools and get tax incentives • No policies against unhealthy products as they may hurt the economy • Nutrition not a priority for policymakers compared to topics such as sexual health • Farmers facing difficulties in gaining certificates to participate in school feeding • Slow translation of international policies and guidelines into national • Schools for feeding programs chosen based on votes of the region for certain political parties	• Legislation incentivized purchases from farmers • Education government sector taking the lead in introducing nutrition-related programs for children from a poor background • Health government sector taking the lead in promoting school health • Decentralization of meal program enabled the participation of local farmers • Various legislation enabled the participation of local farmers in school feeding • When nutritional security is a priority at the national level, inter-sectoral cooperation and new legislation in this direction introduced • When international programs complementary to national programs, enabled the sustainability of national programs
Inner setting	Structural characteristics		Number of articles: 14	Number of articles: 2
			• Lack of space for food preparation; lack of a kitchen • Lack of eating area • No food storage facilities • Problematic school structure; small schools • Lack of water and light	• Private schools have more land for planting crops • International bodies (World Food Programme) providing storage boxes • Vegetable gardens on school grounds
	Readiness for implementation	Available resources	Number of articles: 23	Number of articles: 4
			• Not enough teachers, canteen workers, and external professionals to help with nutrition-related interventions/programs/policies • Capacity building, training needed for various activities (e.g., using school meal planner tools, training for teachers on the management of nutrition program, training for cooks) • No financial resources for some actions (e.g., educational activities) • Rent paid by kiosks (serving unhealthy food) on school property used for different school needs; profit from canteens used for school financial needs (e.g., teacher salaries); school resources coming from industry • Lack of time of the school staff and children (e.g., teachers and children cleaning up, teachers having to attend after-school meetings, monitoring by principals, teaching and nutrition coordination by teachers, eating time for fruit and vegetables) • No training materials • Lack of equipment such as scales and blenders	• Workshops and training provided (e.g., for lunch ladies, cooks, nutrition education for teachers with no such background, instruction on how to incorporate nutrition in the curriculum) • Materials for teachers easing integration of interventions in the curriculum
Characteristics of individuals	Knowledge and beliefs about the scheme		Number of articles: 8	Number of articles: 14
			• Principals’ and teachers’ belief that children do not like healthy food, fruit, and vegetables • Teachers’ belief that children do not have an understanding about healthy eating • Principals’ belief that family habits shape what children eat, and it is the family’s responsibility to make sure they eat healthy • Teachers belief that parents do not provide nutrition-rich food and lack knowledge of the topic • Lack of knowledge among the school staff (principals, kiosks workers) on existing policies, guidelines as well as nutrition more generally	• School staff’s (principals, pedagogical coordinators) belief that it is possible to change child eating habits in the school setting and that it is the responsibility of the school to do so • School staff’s belief that nutrition food is good for the children and school meal programs are good for the entire community • School staff’s belief that school feeding programs increase attendance and improve grades and discipline • Principals’ and teachers’ belief that school programs can positively change eating habits at home and children may carry them into adulthood • Teachers’ belief that school feeding is a basic human right • Nutrition intervention motivating teachers to improve their own health
Process	Engaging	External change agents	Number of articles: 16	Number of articles: 8
			• Funds given to cooperatives regardless of work done or problematic documentation submitted • Training to cooperatives limited and without follow-up • No training for family farmers, volunteer cooks, and community members; not informed of the intervention/policy/program properly • When community members involved in feeding programs on a voluntary basis, lack of participation due to challenges with transportation, training, and time • Lack of nutritionists (with the role of monitoring planning execution of school feeding programs); work overload for those nutritionists that were available • National government-level actors (e.g., supervisors from the Ministry of Education) not aware of the roles and responsibilities • Lack of cooperation between government bodies (e.g., health and education) in implementing interventions/policies/programs; each passing the responsibility to the other • Strong industry lobby against any action toward limiting unhealthy food access for children	• When nutritionists available, their role was important in the implementation • Intersectorality (cooperation between different government bodies—e.g., health, education) and cooperation with family farmers • Training for family farmers, cooperatives; family farmers organizing in cooperatives; lobbying for participation in school feeding programs • Advocacy groups with a focus on healthy lifestyles important to bring policy changes • Prioritization of nutrition by government bodies (e.g., education) • Volunteering by community members to contribute to school feeding programs (e.g., members of women’s association preparing meals, village leaders, and midwives monitoring implementation)
		Innovation participants	Number of articles: 10	Number of articles: 17
			• Children expressed views that healthy food was unappealing and lacked variety • Low participation by children in activities taking place after school hours • Parents informed of interventions/policies/programs through notes, their participation in turn low • Parents not informed of the interventions/policies/programs; not involved in decision-making at the national level and in some cases school level • Generally lack of involvement of parents; when activities available, challenges to participate due to work obligations • Parents perceived vegetables as something you eat when you are poor	• Encouragement, guidance, and motivation of children by teachers during meals; discussing food waste • Children looking forward to school due to the meal; children coming to school early due to breakfast being served • Children with a positive impression of healthy food; belief it is good for health, mood, weight control, and concentration • Educational, group activities and lectures for children and parents (food and nutrition fairs for parents and children) • Child involvement in developing materials; children as change agents for the program; promoting hygiene practices • Parents given a chance to participate in mid-day meal program; invited to cooking competitions; invited to taste the school meal food • Parents’ outreach through social media • Nutritionists speaking to parents seen as helpful • Health experts invited to parent meetings • Positive views toward interventions/policies/programs in particular by parents of a poor background • Improvements in hygiene perceived as a result of intervention/policy/program • Improvement of eating practices at home
	Executing		Number of articles: 11	Number of articles: 2
			• Late, irregular payment by the government to cooperatives, suppliers, caterers, and farmers • Delayed submission of documentation by principals, delaying payments to suppliers • Various difficulties with the delivery of products, including insufficient quantities • Late deliveries of cooking gas • No enforcement of food policy by principals, so as not to decrease the profit of on-site sellers • No visits by nutritionists implemented • Food committee members responsible for monitoring did not implement visits • Some funds embezzlement by food committee members	• Some positive changes in schools in regard to selling unhealthy foods following food policy at the national level • Hygiene practices implemented as a result of home-grown school feeding program

For an overview of all constructs found within the included articles, please see Additional file 7. For a summary of the barriers and facilitators found under each construct/subconstruct across the included articles please see Additional file 3.

#### Domain 1: Intervention characteristics

The most widely prevalent constructs from the *intervention characteristics* domain were *design quality and packaging* and *cost*. *Design quality and packaging* was identified in 20 papers overall [27, 29, 31–34, 36, 39–42, 44, 45, 53, 54, 57–61], in 15 as a barrier and in 10 as a facilitator. The most commonly identified barriers were linked to the diversity, quality, and quantity of the products or food provided in school settings. For example, repetitive menus, or menus which were not taking into consideration dietary limitations such as allergies, as well as cultural diversity, were identified as a barrier. Articles also identified the lack of fruit and vegetables provided in schools as a barrier. Overall, the quantity of the products or food provided was not enough to serve all children in the schools. The quality criteria for the products and food provided were found to be lacking, and in some articles, it was noted that the food was generally of low quality, and the use of agro-chemicals was of concern. A lack of diversity of activities was also identified as a barrier in regard to nutrition education-related action. Finally, the unappealing taste and appearance of healthy food and food waste were also identified as barriers.

On the contrary, the provision of fresh products of good quality and nutritional value was identified as a facilitator to the implementation of the various actions. The use of homemade food, local products, and products with less chemicals due to organic fertilizers being used were identified as facilitators. It was a facilitator when a variety of snacks were available to children, and the cooking of meals was based on set, diverse menus. Finally, in regard to nutrition-related education actions in particular, it was identified as a facilitator when health education was provided to children, and information was provided to teachers in regard to the damage that sugar-sweetened beverages and processed foods cause. The use of videos and diverse educational activities as part of these types of actions was a facilitator.

*Cost* was identified across 12 papers [27, 28, 34, 36, 45, 51, 52, 54, 56, 58–60], predominantly as a barrier (11 papers), and a facilitator in one paper. Overall, several papers identified the high cost of healthy food as a barrier. The short shelf life of healthy food was one of the factors which may influence the higher price. Some papers pointed specifically to the high cost of fruit and vegetables, homemade food, and food that came from local farmers, all of higher price as they are not mass produced, as a barrier. The funds allocated to schools were insufficient for the purchase of nutritionally rich food as well as not sufficient to provide for all the children in the schools. In some articles, it was noted that externally provided funds were not adjusted after inflation, nor following economic crises which increased food price. Cost was identified as a facilitator when in addition to the government, farmers and cooperatives also invested their own funds to be able to participate in food provision programs, such as purchasing of trucks to deliver the food to schools.

#### Domain 2: Outer setting

The most widely prevalent constructs of the *outer setting* domain were *target group needs and resources*, *cosmopolitanism*, and *external policy and incentives*. *Target group needs and resources* were identified across 13 papers [15, 29, 31, 32, 37, 38, 40, 42, 44, 46, 54, 58, 59], in 8 as a facilitator and in 5 as a barrier. As already reported, 14 out of the 36 papers included in this review clearly stated that the studied action was targeting children in areas of low socio-economic background (see Additional file 5). With this focus, the perception that school meals contributed to fulfilling the nutritional needs of children in poor communities was a facilitator. It was expressed that for some children, the school meal may be the only full meal of the day and a reason why they may be sent to school, which increased attendance rates. In addition, free school meals meant that parents do not have to give snack money to their children, thus affording some savings for the family. The children would also be less able to buy unhealthy food available in and around school settings. When school meal leftovers were sent home to benefit the family, this was also identified as a facilitator. Purchasing food from local farmers was a facilitator as it economically helped the community. Finally, schools that afforded access to health and nutrition-related services to children of lower socio-economic background was a facilitator.

However, when healthy food was not provided for free, the school opted for making more affordable, unhealthy food available and this was a barrier. In addition, when parents were responsible for contributing toward school meals, children most in need were excluded which was a barrier. When food was distributed to all equally regardless of individual parental contributions, this was found demotivating for those parents that did contribute and, thus, a barrier.

*Cosmopolitanism* was identified across 18 papers [28, 30, 32, 37, 39, 42–44, 47, 49, 51–54, 56, 57, 59, 61], as a barrier in 11 and a facilitator in 8. The dominant barrier identified across papers was the challenging interaction between actors of the school setting (e.g., principals, teachers) and those external to the school setting (e.g., cooperatives and suppliers, caterers, community members). Principals expressed the frustration that cooperatives in particular could be unreliable, not delivering food on time, and not being able to handle the administrative requirements for participation in meal programs. A lack of cooperation between schools when implementing similar actions was also identified as a barrier. When schools formed partnerships with industry, where the school would receive funding in exchange for allowing the presence of unhealthy food products on school grounds, this was an important barrier. Finally, not including schools in the decision-making process by the educational sector of the government in particular was also a barrier.

When schools cooperated with food kiosks, on specific obesity prevention programs, this was a facilitator. Overall, when good cooperation was established between the school and various community actors such as farmers, cooperatives, and various government bodies from the health and education sectors, this was a facilitator. For example, in one instance, local women volunteered to cook the school meals, and as a benefit could take home any leftovers, which was a facilitator for that particular food provision program [59].

*External policy and incentives* were identified across 14 papers [32, 34, 36, 38, 40, 43, 51, 52, 54–56, 58–60], in 12 as a barrier and 5 as a facilitator. One of the dominant barriers found was the lack of legal sanctions or consequences for schools, when not complying with a particular program or policy. Principals also complained about poor information in regard to new national nutrition-related actions. They were informed either through a short notice or they simply had to find out on their own by reading the news. When this happened, implementation guidance and funding were also lacking, which was an additional barrier. Industry lobbying against any actions which would limit the presence of sugar-sweetened beverages and unhealthy food in school settings was identified as an important barrier. Even further, in some contexts, policies were in place that allowed industry actors to receive tax incentives when donating funds to schools, in return for which they could promote their products in school settings. The slow translation of international actions into national legislation was also a barrier.

In cases where legislation was in place that would ease the purchase from local farmers, this was a facilitator. Decentralization of meal programs, facilitating the use of local food, was one such example. The education and health sectors of the government taking the lead in promoting nutrition-related actions for children of lower socio-economic background, in particular, was also a facilitator. In this direction, it was a facilitator when nutrition was identified as a priority at the national level, which further encouraged inter-sectoral cooperation. Finally, when international actions were found to be complementary to those at the national level, this was a facilitator that contributed toward the sustainability of those actions.

#### Domain 3: Inner setting

The most widely prevalent constructs/subconstructs of the *inner setting* domain were *structural characteristics* and *available resources* (part of readiness for implementation construct). *Structural characteristics* was identified across 16 papers [15, 32, 36, 37, 39–43, 53, 54, 56–58, 60, 61], as a barrier in 14 and a facilitator in two papers. Most commonly, the lack of space for food preparation (e.g., lack of a kitchen) and eating were identified as barriers, as well as not having running or clean water and electricity. In addition, schools did not have storage space for food which was a barrier. Finally, problems with the school structure overall and the schools being too small were also identified as barriers.

On the other hand, schools having more space and thus the possibility for planting crops and having gardens on school property were identified as a facilitator. The provision of storage boxes by international bodies (e.g., World Food Programme) was also identified as a facilitator.

The subconstruct *available resources* was the most prevalent across domains, identified in a total of 24 papers [15, 27, 32–35, 38–44, 46–48, 50–54, 56, 57, 61], as a barrier in 23 and a facilitator in four. Not having enough staff (e.g., teachers, canteen workers, external professionals such as nutritionists) was a barrier. For the staff that was available, lack of time to dedicate to nutrition-related actions was a barrier. For example, it was difficult for the small number of teachers relative to the number of pupils, to split their time between in-class activities, and nutrition-related activities that could also include after-school meetings. Principals found it equally difficult to incorporate monitoring activities of food provision within their usual daily schedules. Lack of training and capacity-building activities for various staff (teachers, cooks) was identified as another barrier. Finally, the lack of financial resources by the schools more generally was also emphasized. In this regard, some schools received part of their financial resources from industry, or by renting out space to kiosks and vendors, and from the profit made by canteens. In these cases, the school staff was more tolerant to the promotion or presence of unhealthy food so as to ensure the inflow of funding, and this was thus a barrier to nutrition-related actions.

When workshops or training was provided for various staff (cooks, lunch ladies, nutrition education for teachers), this was found to be a facilitator. In addition, making materials available to teachers that would ease the integration of nutrition-related activities into the regular curriculum was also a facilitator.

#### Domain 4: Characteristics of individuals

From the *characteristics of individuals* domain, the most prevalent construct was *knowledge and beliefs* of the school staff (principals, teachers, pedagogic coordinators). The construct was identified across 20 papers [15, 27, 29, 31, 32, 36, 38, 40, 42–44, 47, 50–52, 54, 56, 57, 60], a facilitator in 14 and a barrier in eight. The belief by principals and teachers in that school feeding programs improved attendance, grades, and discipline was a commonly identified facilitator. It was also a facilitator when the school staff believed it is the responsibility of the school to provide healthy food for the children. In addition, beliefs that the healthy habits created at school may be transmitted to the home setting and carried into adulthood were also facilitators.

When principals, teachers, and other school staff expressed the view that children do not like healthy food, this was a barrier. It was a barrier when teachers expressed the view that it was not the responsibility of the school to provide healthy food, while at the same time, they said that children are not being fed healthy food at home. Overall, the lack of knowledge of the school staff (principals, kiosks workers, teachers) in regard to nutrition more generally, or to existing food and nutrition policies and guidelines in particular (e.g., no understanding of the criteria used to determine if some food products were allowed or not allowed for sale in food kiosks; belief that packaged chips which are baked and not fried are allowed), was also a barrier.

#### Domain 5: Process

The most widely prevalent constructs/subconstructs of the *process* domain were *external change agents*, *innovation participants* (part of the engaging construct), and *executing*. *External change agents* was identified across 19 papers [27, 28, 31, 32, 34, 37, 43–45, 51–60], as a barrier in 16 and a facilitator in eight. The identified barriers had to do with four groups of actors whose engagement was challenging: cooperatives/family farmers/suppliers, government employees (e.g., health, education), community members, and industry. In regard to cooperatives, the perception was shared that they receive funds, regardless of the quality of their service. Cooperatives, family farmers, and suppliers also often did not receive training or training was insufficient which was a barrier. There was a lack of nutritionists needed for some meal programs, while overall, government employees were found to lack awareness of their roles and did not cooperate with each other which was a barrier. Although community members volunteered to participate in some programs, they often did not fulfill their responsibilities due to challenges with transportation, time, and lack of training. Finally, the active involvement of industry lobbying against policies limiting unhealthy food was a barrier.

On the contrary, when training was offered to family farmers, nutritionists were available, and good cooperation existed between government bodies in the implementation process, these were all identified as facilitators. Engagement of advocacy groups in favor of healthy eating and lifestyles was also a facilitator.

*Innovation participants* was identified across 23 papers [15, 27, 29, 30, 32–36, 40, 42, 44, 46–50, 54, 57–59, 61], as a facilitator in 17 and a barrier in 10. Overall, when children expressed positive views toward healthy food, were further motivated to eat healthily by teachers, and considered the meal as an important reason to attend school, these were all facilitators. Organized activities for both children and parents and their active inclusion in nutrition-related, school-based actions was a facilitator. Examples of such activities are using children as change agents, parents in the preparation of meals, inviting parents to cooking competitions, and inviting parents to taste the school meals. The invitation of nutritionists and other health experts to directly speak to parents was also a facilitator promoting parental engagement. Some parents expressed the view that nutrition-related school initiatives improved eating practices at home.

When children found healthy food as unappealing and lacking variety, this was a barrier. Child participation in program activities outside of school hours was also a barrier, as was a lack of parental participation in some school-based nutrition related-activities, as they did not have the time.

*Executing* was identified across 12 papers [31, 32, 38, 39, 42, 45, 51, 53, 54, 57, 59, 60] as a barrier in 11 and a facilitator in two. One of the most common barriers stated in regard to executing was the late and irregular payment of government funds to suppliers/cooperatives/caterers/family farmers. In some cases, this was linked to delayed submission of necessary documentation by those receiving the funds and the principals of schools. This in turn caused delays in the delivery of food and in some cases insufficient quantity, which were additional barriers. The lack of enforcement of healthy food policies by principals so as not to influence the profit of school-based sellers was also a barrier. When policies were implemented properly, be it in regard to limiting the presence of unhealthy food on school grounds or ensuring proper hygiene practices, this was a facilitator.

## Discussion

This review filled a gap in the literature by studying barriers and facilitators to the implementation of nutrition-related actions in school settings in LMICs. We identified barriers and facilitators to implementation linked to the following CFIR constructs/sub-constructs: design quality and packaging, cost (intervention characteristics); target group needs and resources, cosmopolitanism, external policy and incentives (outer setting); structural characteristics, readiness for implementation (inner setting); knowledge and beliefs (characteristics of individuals) and engaging, executing (process). The outer setting is the only domain where most of the constructs (three out of four) were present across at least 12 papers.

Research both in- and outside of LMIC contexts and in- and outside of school settings highlights the relevance of constructs such as target group needs and resources [62, 63], knowledge and beliefs [16, 64], and engagement [65, 66]. Many of the included papers were linked to actions that were specifically targeting areas of low socio-economic background, which may explain why awareness of target group (child and family) needs and resources was particularly highlighted as a facilitator in school settings. In fact, it is notable that most of the constructs present were barriers, except those having to do with the needs and views of children and implementers within the schools (target group needs and resources, knowledge and beliefs, innovation participants) which were predominantly facilitators. This means that in addition to the needs of children and their families being clearly recognized, having school staff who firmly believe in the actions and are willing to engage with the target group to further promote those actions is important. Consistent with our findings, a meta-review studying CFIR-based constructs relevant to implementing healthy diet/physical activity/sedentary behavior policies in school settings found the importance of knowledge and beliefs highlighted in nine out of ten reviews included in the analysis [67]. Similarly, a scoping review studying the factors that influence the implementation of nutrition policies in schools internationally stressed the relevance of having a common purpose and responsibility among stakeholders within the school setting, as well as belief in and acceptance of a policy by stakeholders such as teachers [17].

On the other hand, some of the predominant barriers identified were cost of the actions, structural characteristics, lack of resources of the schools, cosmopolitanism, external policy and incentives, and external change agents. In other research, whereas cost [16], structural characteristics [65], and lack of resources [16, 63, 65, 68] are also mostly barriers, cosmopolitanism [16, 62, 64, 65, 69, 70], external policy and incentives [16, 69, 70], and external change agents [16, 71] can be found as both barriers and facilitators. We found that schools consistently faced challenges when it came to financial and other resources, and one way of addressing this was by making their premises available at a cost to external actors and food industry whose interests were rarely aligned with the nutrition-related actions in place. That the government actors faced their own troubles not only in regard to cooperation among each other (health, education) but also in regard to informing and supporting schools in regard to existing initiatives, as well as combating industry interests may just be a further reinforcing factor of all of the above-stated constructs as barriers. Sobers and colleagues found similar barriers as those in our review, in their study of the implementation of nutrition guidelines in schools in Barbados, highlighting poor dissemination from the government level, low engagement of the educational sector of the government, and the presence of vending machines and school cafeterias which share profits with the school on school grounds [72]. The authors call for a whole of society approach with stronger cooperation across all government sectors and stakeholders involved to deal with these challenges [72]. However, in practice, the lack of inter-sectoral cooperation is an important barrier found in our research, as well as elsewhere [16]. McIsaac and colleagues also highlight the significance of macro-level support in nutrition policy implementation in schools internationally, such as clarity of policy execution and language, as well as training support and resources [17]. The same review identified financial aspects such as cost of healthy food, revenue and profit margins for cafeterias and canteens, and school fundraising as significant barriers [17].

### Recommendations

Based on the findings of our review, we have the following recommendations:

- When introducing nutrition-related actions, efforts should be made at the government level to ensure that these are well understood by those adopting and implementing the policies in the school setting, such as principals and teachers. Furthermore, implementation assistance (know-how and/or financial) should be offered if needed.
- Free provision of healthy food to children is not only beneficial in terms of savings for the family, but also, the children have less possibilities to buy unhealthy food (if they are not given snack money), and thus, there is less incentive to make unhealthy food available, be it by vendors outside or inside the school.
- As schools are left dependent on external actors, such as industry, vendors, kiosk, and cafeteria operators for their finances, nutrition-related priorities may be neglected. Providing schools with the necessary resources to prevent this dependency on external actors is key. These can be for example government provision of financial aid or introduction of policies which would limit the profit activities on school grounds.
- Cooperation among external (government, suppliers, community) and internal (principals, teachers, children) actors in the school setting is key, and as such, more efforts toward inter-sectoral interactions as well as various activities such as training targeted to the specific stakeholders and their interactions should be considered.
- Finally, a recommendation linked to future research on this topic is to take advantage of and apply available theoretical frameworks (such as the CFIR), which would ensure not only clarity of findings considering the consistent use of terminology relevant to practitioners and academia alike, but also comparability within and across disciplines.

### Strengths and limitations

A systematic method, from the search to the end of the analysis process, using a widely applied framework from the field of implementation science (the CFIR) was followed. However, this work also has some shortcomings. The CFIR was not used in the included studies; thus, the coding and identification of barriers and facilitators under the different constructs of the framework were done by the authors. Although the articles included were of mixed quality, we decided not to exclude any of them on the basis of the quality assessment, as the findings were evaluated to be of relevance. There is more research done on certain types of actions in particular countries (e.g., school meals in Brazil), less on others (e.g., environmental change actions). This is reflected in the number of articles focusing on school meals, particularly from Brazil, which has been included in our review. In this regard, although our review filled a knowledge gap regarding the implementation of all types of nutrition-related actions in school settings across all LMICs, it may be beneficial for future research to distinguish between different actions when studying barriers and facilitators to implementation, such as a focus on school meals only. Finally, we reported on the identified barriers and facilitators based on prevalence across the included papers (even though all barriers and facilitators found are reported in Additional file 3); however, we did not explore the relationships between different constructs as the data was not sufficient to make this kind of analysis.

## Conclusion

This review has identified barriers and facilitators to the implementation of nutrition-related actions based on qualitative articles in the school setting in LMICs, using the CFIR. We found that most constructs prevalent across papers were predominantly barriers, with the exception of target group needs and resources, knowledge and beliefs, and innovation participants.

## Supplementary Information

**Additional file 1.** Documentation of search.

**Additional file 2.** Framework matrix.

**Additional file 3.** Summary of barriers and facilitators.

**Additional file 4.** ENTREQ Checklist.

**Additional file 5.** Characteristics of articles.

**Additional file 6.** Quality assessments.

**Additional file 7.** Constructs by study.

## Data Availability

All data analyzed during this study are included in this published article [and its supplementary information files].

## Abbreviations

NCDs: Non-communicable diseases
LMICs: Low- and middle-income countries
WHO: World Health Organization
CFIR: Consolidated Framework for Implementation Research
CASP: Critical Appraisal Skills Programme
