# Supplementary material for: Barriers and facilitators to implementation of nutrition-related actions in school settings in low- and middle-income countries (LMICs): a qualitative systematic review using the Consolidated Framework for Implementation Research (CFIR)
Source: Implement Sci Commun. 2023 Jun 27;4:73. doi: 10.1186/s43058-023-00454-y (PMC10294384; doi:10.1186/s43058-023-00454-y)
Supplement: Supplementary file 1 — Additional file 1. Documentation of search. [file 43058_2023_454_MOESM1_ESM.docx]

**Search report: Double duty action (nutrition related actions) implementation review**

**Ovid Embase, Medline, ERIC, Global Healht, Medline, PsycInfo**

Embase Classic+Embase 1947 to 2021 October 12, ERIC 1965 to May 2021, Global Health 1973 to 2021 Week 39, Ovid MEDLINE(R) ALL 1946 to October 12, 2021, APA PsycInfo 1806 to October Week 1 2021.

**Date of search:** October 13th 2021. **Number of retrieved references:** Embase 879, ERIC 130, Global Health 1070, Medline 688, PsycInfo 203

| 1 | (((nutrition or diet or dietary or food or obesity or overweight or physical* activ* or physical* inactiv* or sport? or exercise? or sedentary or "active travel" or "active transport*" or "active commut*" or "transport mode" or "transportation mode" or "travel mode" or cycle or cycling or bicycle or bicycling or cyclist* or walking or walk*) adj3 (intervention? or program* or policies or policy or promotion or campaign?)) or double-duty action?).tw,kw,kf. | 275898 |
| --- | --- | --- |
| 2 | (Nutrition Policy/ or ((Sedentary Behavior/ or exp Exercise/ or Bicycling/ or exp walking/ or exp Transportation/) and Health policy/)) use medall | 11015 |
| 3 | (nutrition policy/ or ((physical activity/ or physical inactivity/ or sedentary lifestyle/ or walking/ or cycling/ or exp sport/ or "traffic and transport"/) and health care policy/)) use emczd | 5328 |
| 4 | (((Nutrition/ or Food/ or Dietetics/ or Obesty/) and (Health Programs/ or Health Promotion/)) or (Food Standards/ or Nutrition Instruction/ or Breakfast Programs/ or Lunch Programs/)) use eric | 5702 |
| 5 | ((exp Nutrition/ or exp Food/ or exp Physical Activity/ or exp Exercise/ or Sports/ or Physical fitness/ or overweight/ or obesity/ or exp Sedentary Behavior/) and (exp Intervention/ or health promotion/ or public health campaigns/)) use psyh | 13975 |
| 6 | (nutritional intervention/ or nutrition programmes/ or ((nutrition/ or food/ or diet/ or physical activity/ or exercise/ or walking/ or overweight/ or obesity/ or exercise/ or bicycling/ or sport/ or transport/) and (health programmes/ or health promotion/))) use cagf | 20966 |
| 7 | or/1-6 | 308930 |
| 8 | (implement* or barrier* or facilitator* or determinant?).tw,kw,kf. | 3367847 |
| 9 | (Health Plan Implementation/ or Implementation Science/) use medall | 7394 |
| 10 | implementation science/ use emczd | 2218 |
| 11 | Program Implementation/ use eric | 27032 |
| 12 | ("implementation of research"/ or project implementation/) use cagf | 972 |
| 13 | or/8-12 | 3369886 |
| 14 | (nursery or nurseries or kindergarten? or kindergarden? or pre-school? or preschool? or school? or junior high? or high-school? or elementary-school? or junior-high? or middle-school? or class or classes or classroom? or pupil? or teacher?).tw,kw,kf. | 4042582 |
| 15 | (Schools, Nursery/ or schools/) use medall | 45072 |
| 16 | (school/ or high school/ or kindergarten/ or middle school/ or primary school/) use emczd | 115976 |
| 17 | (classrooms/ or "classes (groups of students)"/ or exp schools/ or Kindergarten/ or preschools/) use eric | 272896 |
| 18 | (schools/ or elementary schools/ or high schools/ or junior high schools/ or kindergartens/ or middle schools/ or nursery schools/ or classrooms/) use psyh | 69656 |
| 19 | (school children/ or schools/ or elementary schools/ or high schools/ or nursery schools/ or teachers/ or pupils/) use cagf | 40244 |
| 20 | or/14-19 | 4105968 |
| 21 | (afghanistan or albania or algeria or american samoa or angola or "antigua and barbuda" or antigua or barbuda or argentina or armenia or armenian or aruba or azerbaijan or bahrain or bangladesh or barbados or republic of belarus or belarus or byelarus or belorussia or byelorussian or belize or british honduras or benin or dahomey or bhutan or bolivia or "bosnia and herzegovina" or bosnia or herzegovina or botswana or bechuanaland or brazil or brasil or bulgaria or burkina faso or burkina fasso or upper volta or burundi or urundi or cabo verde or cape verde or cambodia or kampuchea or khmer republic or cameroon or cameron or cameroun or central african republic or ubangi shari or chad or chile or china or colombia or comoros or comoro islands or iles comores or mayotte or democratic republic of the congo or democratic republic congo or congo or zaire or costa rica or "cote d’ivoire" or "cote d’ ivoire" or cote divoire or cote d ivoire or ivory coast or croatia or cuba or cyprus or czech republic or czechoslovakia or djibouti or french somaliland or dominica or dominican republic or ecuador or egypt or united arab republic or el salvador or equatorial guinea or spanish guinea or eritrea or estonia or eswatini or swaziland or ethiopia or fiji or gabon or gabonese republic or gambia or "georgia (republic)" or georgian or ghana or gold coast or gibraltar or greece or grenada or guam or guatemala or guinea or guinea bissau or guyana or british guiana or haiti or hispaniola or honduras or hungary or india or indonesia or timor or iran or iraq or isle of man or jamaica or jordan or kazakhstan or kazakh or kenya or "democratic people’s republic of korea" or republic of korea or north korea or south korea or korea or kosovo or kyrgyzstan or kirghizia or kirgizstan or kyrgyz republic or kirghiz or laos or lao pdr or "lao people's democratic republic" or latvia or lebanon or lebanese republic or lesotho or basutoland or liberia or libya or libyan arab jamahiriya or lithuania or macau or macao or republic of north macedonia or macedonia or madagascar or malagasy republic or malawi or nyasaland or malaysia or malay federation or malaya federation or maldives or indian ocean islands or indian ocean or mali or malta or micronesia or federated states of micronesia or kiribati or marshall islands or nauru or northern mariana islands or palau or tuvalu or mauritania or mauritius or mexico or moldova or moldovian or mongolia or montenegro or morocco or ifni or mozambique or portuguese east africa or myanmar or burma or namibia or nepal or netherlands antilles or nicaragua or niger or nigeria or oman or muscat or pakistan or panama or papua new guinea or new guinea or paraguay or peru or philippines or philipines or phillipines or phillippines or poland or "polish people's republic" or portugal or portuguese republic or puerto rico or romania or russia or russian federation or ussr or soviet union or union of soviet socialist republics or rwanda or ruanda or samoa or pacific islands or polynesia or samoan islands or navigator island or navigator islands or "sao tome and principe" or saudi arabia or senegal or serbia or seychelles or sierra leone or slovakia or slovak republic or slovenia or melanesia or solomon island or solomon islands or norfolk island or norfolk islands or somalia or south africa or south sudan or sri lanka or ceylon or "saint kitts and nevis" or "st. kitts and nevis" or saint lucia or "st. lucia" or "saint vincent and the grenadines" or saint vincent or "st. vincent" or grenadines or sudan or suriname or surinam or dutch guiana or netherlands guiana or syria or syrian arab republic or tajikistan or tadjikistan or tadzhikistan or tadzhik or tanzania or tanganyika or thailand or siam or timor leste or east timor or togo or togolese republic or tonga or "trinidad and tobago" or trinidad or tobago or tunisia or turkey or turkmenistan or turkmen or uganda or ukraine or uruguay or uzbekistan or uzbek or vanuatu or new hebrides or venezuela or vietnam or viet nam or middle east or west bank or gaza or palestine or yemen or yugoslavia or zambia or zimbabwe or northern rhodesia or global south or africa south of the sahara or sub-saharan africa or subsaharan africa or africa, central or central africa or africa, northern or north africa or northern africa or magreb or maghrib or sahara or africa, southern or southern africa or africa, eastern or east africa or eastern africa or africa, western or west africa or western africa or west indies or indian ocean islands or caribbean or central america or latin america or "south and central america" or south america or asia, central or central asia or asia, northern or north asia or northern asia or asia, southeastern or southeastern asia or south eastern asia or southeast asia or south east asia or asia, western or western asia or europe, eastern or east europe or eastern europe or developing country or developing countries or developing nation? or developing population? or developing world or less developed countr* or less developed nation? or less developed population? or less developed world or lesser developed countr* or lesser developed nation? or lesser developed population? or lesser developed world or under developed countr* or under developed nation? or under developed population? or under developed world or underdeveloped countr* or underdeveloped nation? or underdeveloped population? or underdeveloped world or middle income countr* or middle income nation? or middle income population? or low income countr* or low income nation? or low income population? or lower income countr* or lower income nation? or lower income population? or underserved countr* or underserved nation? or underserved population? or underserved world or under served countr* or under served nation? or under served population? or under served world or deprived countr* or deprived nation? or deprived population? or deprived world or poor countr* or poor nation? or poor population? or poor world or poorer countr* or poorer nation? or poorer population? or poorer world or developing econom* or less developed econom* or lesser developed econom* or under developed econom* or underdeveloped econom* or middle income econom* or low income econom* or lower income econom* or low gdp or low gnp or low gross domestic or low gross national or lower gdp or lower gnp or lower gross domestic or lower gross national or lmic or lmics or third world or lami countr* or transitional countr* or emerging economies or emerging nation?).ti,ab,sh,kf,kw. | 6233084 |
| 22 | (afghan or afghans or afghani or albanian? algerian? or american samoan? or angolan? or antiguan? or barbudan? or argentine? or argentinian? or argentinean? or armenian? or aruban? or azerbaijani? or bahraini? or bangladeshi? or bangalees or bajan? or belarusian? or byelorussian? or belizean? or beninese? or bhutanese or bolivian? or bosnian? or botswana or batswana or brazilian? or brasilian? or bulgarian? or burkinabe or burkinese or burundian? or cape verdean? or cabo verdean? or cambodian? or khmer or cameroonian? or central african? or chadian? or chilean? or chinese or colombian? or comorian? or congolese or costa rican? or ivorian? or croatian? or cuban? or cypriot? or czech? or djiboutian? or dominican? or ecuadorian? or egyptian? or salvadoran? or equatorial guinean? or equatoguinean? or eritrean? or estonian? or swazi? or swati? or ethiopian? or fijian or gabonese or gabonaise or gambian? or georgian? or ghanaian? or gibraltarian? or greek? or grenadian? or guamanian? or guatemalan? or guinean? or bissau guinean? or guyanese or haitian? or honduran? or hungarian? or indian? or indonesian? or iranian? or iraqian? or iraqi? or manx or jamaican? or jordanian? or kazakhstani? or kenyan? or kirabati or kirabatian? or north korean? or korean? or kosovar? or kosovan? or kyrgyz* or lao or laotian? or latvian? or lebanese or lesothan? or lesothonian? or mosotho or basotho or liberian? or libyan? or lithuanian? or macanese or macedonian? or malagasy or madagascan? or malawian? or malaysian? or maldivian? or malian? or maltese or marshallese? or mauritanian? or mauritian? or mexican? or micronesian? or moldovan? or mongolian? or mongol or montenegrin? or moroccan? or mozambican? or burmese or myanma or namibian? or nauruan? or nepali or nepalese or netherlands antillean? or nicaraguan? or nigerien? or nigerian? or northern mariana islander? or mariana? or omani? or pakistani? or palauan? or panamanian? or papua new guinean? or paraguayan? or peruvian? or philippine? or philipine? or phillipine? or phillippine? or filipino? or filipina? or polish or pole or poles or portuguese or puerto rican? or romanian? or russian? or soviet people or soviet population or rwandan? or rwandese or ruandan? or ruandese or samoan? or sao tomean? or santomean? or saudi arabian? or saudi? or senegalese or serbian? or montenegrin? or seychellois or seychelloise? or sierra leonean? or slovak? or slovene? or solomon islander? or somali? or south african? or south sudanese or sri lankan? or ceylonese or kittitian? or nevisian? or saint lucian? or vincentian? or sudanese or surinamese? or syrian? or tajik? or tajikistani? or tanzanian? or tanganyikan? or thai or timorese? or togolese or tongan? or trinidadian? or tobagonian? or tunisian? or turk? or turkish or turkmen? or tuvaluan? or ugandan? or ukrainian? or uruguayan? or uzbek? or vanuatu* or venezuelan? or vietnamese or yemeni? or yemenite? or yemenese or yugoslav? or yugoslavian? or zambian? or zimbabwean?).ti,ab,sh,kf,kw. | 2930992 |
| 23 | or/21-22 | 7619200 |
| 24 | 7 and 13 and 20 and 23 | 2970 |
|  | Embase | 879 |
|  | ERIC | 130 |
|  | Global Health | 1070 |
|  | Medline | 688 |
|  | PsycInfo | 203 |
| 25 | remove duplicates from 24 | 1998 |
| 26 | 25 use medall | 686 |
| 27 | 25 use emczd | 373 |
| 28 | 25 use eric | 130 |
| 29 | 25 use psyh | 96 |
| 30 | 25 use cagf | 713 |

Key: psyh= search in PsycInfo, medall=search in Medline, cagf= search in Global Health, emczd=search in Embase, ERIC=search in ERIC. / = subject heading, tw = title, abstract, kw = author supplied keywords in Embase, kf = author supplied keywords in Medline, ADJn = word distance of maximum n words.

**Cochrane Trials**

**Date of search:** October 13th 2021. **Number of retrieved references:** 380

| #1 | (((nutrition or diet or dietary or food or obesity or overweight or physical* activ* or physical* inactiv* or sport? or exercise? or sedentary or "active travel" or "active transport*" or "active commut*" or "transport mode" or "transportation mode" or "travel mode" or cycle or cycling or bicycle or bicycling or cyclist* or walking or walk*) NEAR/3 (intervention? or program* or policies or policy or promotion or campaign?)) or double-duty action?):ti,ab,kw in Trials | 53722 |
| --- | --- | --- |
| #2 | ([mh ^"Nutrition Policy"] OR (([mh ^"Sedentary Behavior"] OR [mh Exercise] OR [mh ^Bicycling] OR [mh walking] OR [mh Transportation]) AND [mh ^"Health policy"])) in Trials | 357 |
| #3 | #1 OR #2 in Trials | 53727 |
| #4 | (implement* OR barrier? OR facilitator? OR determinant?):ti,ab,kw in Trials | 69546 |
| #5 | ([mh ^"Health Plan Implementation"] OR [mh ^"Implementation Science"]) in Trials | 229 |
| #6 | #4 OR #5 in Trials | 69546 |
| #7 | (nursery OR nurseries OR kindergarten? OR kindergarden? OR pre-school? OR preschool? OR school? OR "junior high?" OR high-school? OR elementary-school? OR junior-high? OR middle-school? OR class OR classes OR classroom? OR pupil? OR teacher?):ti,ab,kw in Trials | 121317 |
| #8 | ([mh ^"Schools, Nursery"] OR [mh ^schools]) in Trials | 2231 |
| #9 | #7 OR #8 in Trials | 121317 |
| #10 | (afghanistan OR albania OR algeria OR "american samoa" OR angola OR "antigua and barbuda" OR antigua OR barbuda OR argentina OR armenia OR armenian OR aruba OR azerbaijan OR bahrain OR bangladesh OR barbados OR "republic of belarus" OR belarus OR byelarus OR belorussia OR byelorussian OR belize OR "british honduras" OR benin OR dahomey OR bhutan OR bolivia OR "bosnia and herzegovina" OR bosnia OR herzegovina OR botswana OR bechuanaland OR brazil OR brasil OR bulgaria OR "burkina faso" OR "burkina fasso" OR "upper volta" OR burundi OR urundi OR "cabo verde" OR "cape verde" OR cambodia OR kampuchea OR "khmer republic" OR cameroon OR cameron OR cameroun OR "central african republic" OR "ubangi shari" OR chad OR chile OR china OR colombia OR comoros OR "comoro islands" OR "iles comores" OR mayotte OR "democratic republic of the congo" OR "democratic republic congo" OR congo OR zaire OR "costa rica" OR "cote d’ivoire" OR "cote d’ ivoire" OR "cote divoire" OR "cote d ivoire" OR "ivory coast" OR croatia OR cuba OR cyprus OR "czech republic" OR czechoslovakia OR djibouti OR "french somaliland" OR dominica OR "dominican republic" OR ecuador OR egypt OR "united arab republic" OR "el salvador" OR "equatorial guinea" OR "spanish guinea" OR eritrea OR estonia OR eswatini OR swaziland OR ethiopia OR fiji OR gabon OR "gabonese republic" OR gambia OR "georgia (republic)" OR georgia OR georgian OR ghana OR "gold coast" OR gibraltar OR greece OR grenada OR guam OR guatemala OR guinea OR "guinea bissau" OR guyana OR "british guiana" OR haiti OR hispaniola OR honduras OR hungary OR india OR indonesia OR timor OR iran OR iraq OR "isle of man" OR jamaica OR jordan OR kazakhstan OR kazakh OR kenya OR "democratic people’s republic of korea" OR "republic of korea" OR north korea OR south korea OR korea OR kosovo OR kyrgyzstan OR kirghizia OR kirgizstan OR "kyrgyz republic" OR kirghiz OR laos OR "lao pdr" OR "lao people's democratic republic" OR latvia OR lebanon OR "lebanese republic" OR lesotho OR basutoland OR liberia OR libya OR "libyan arab jamahiriya" OR lithuania OR macau OR macao OR "republic of north macedonia" OR macedonia OR madagascar OR "malagasy republic" OR malawi OR nyasaland OR malaysia OR "malay federation" OR "malaya federation" OR maldives OR "indian ocean islands" OR "indian ocean" OR mali OR malta OR micronesia OR "federated states of micronesia" OR kiribati OR "marshall islands" OR nauru OR "northern mariana islands" OR palau OR tuvalu OR mauritania OR mauritius OR mexico OR moldova OR moldovian OR mongolia OR montenegro OR morocco OR ifni OR mozambique OR "portuguese east africa" OR myanmar OR burma OR namibia OR nepal OR "netherlands antilles" OR nicaragua OR niger OR nigeria OR oman OR muscat OR pakistan OR panama OR "papua new guinea" OR paraguay OR peru OR philippines OR philipines OR phillipines OR phillippines OR poland OR "polish people's republic" OR portugal OR "portuguese republic" OR "puerto rico" OR romania OR russia OR "russian federation" OR ussr OR "soviet union" OR "union of soviet socialist republics" OR rwanda OR ruanda OR samoa OR "pacific islands" OR polynesia OR "samoan islands" OR "navigator island" OR "navigator islands" OR "sao tome and principe" OR "saudi arabia" OR senegal OR serbia OR seychelles OR "sierra leone" OR slovakia OR "slovak republic" OR slovenia OR melanesia OR "solomon island" OR "solomon islands" OR "norfolk island" OR "norfolk islands" OR somalia OR "south africa" OR "south sudan" OR "sri lanka" OR ceylon OR "saint kitts and nevis" OR "st. kitts and nevis" OR "saint lucia" OR "st. lucia" OR "saint vincent and the grenadines" OR "saint vincent" OR "st. vincent" OR grenadines OR sudan OR suriname OR surinam OR "dutch guiana" OR "netherlands guiana" OR syria OR "syrian arab republic" OR tajikistan OR tadjikistan OR tadzhikistan OR tadzhik OR tanzania OR tanganyika OR thailand OR siam OR "timor leste" OR "east timor" OR togo OR "togolese republic" OR tonga OR "trinidad and tobago" OR trinidad OR tobago OR tunisia OR turkey OR turkmenistan OR turkmen OR uganda OR ukraine OR uruguay OR uzbekistan OR uzbek OR vanuatu OR "new hebrides" OR venezuela OR vietnam OR "viet nam" OR "middle east" OR "west bank" OR gaza OR palestine OR yemen OR yugoslavia OR zambia OR zimbabwe OR "northern rhodesia" OR "global south" OR "africa south of the sahara" OR "sub saharan africa" OR "subsaharan africa" OR "africa, central" OR "central africa" OR "africa, northern" OR "north africa" OR "northern africa" OR magreb OR maghrib OR sahara OR "africa, southern" OR "southern africa" OR "africa, eastern" OR "east africa" OR "eastern africa" OR "africa, western" OR "west africa" OR "western africa" OR "west indies" OR "indian ocean islands" OR caribbean OR "central america" OR "latin america" OR "south and central america" OR "south america" OR "asia, central" OR "central asia" OR "asia, northern" OR "north asia" OR "northern asia" OR "asia, southeastern" OR "southeastern asia" OR "south eastern asia" OR "southeast asia" OR "south east asia" OR "asia, western" OR "western asia" OR "europe, eastern" OR "east europe" OR "eastern europe" OR "developing country" OR "developing countries" OR "developing nation" OR "developing nations" OR "developing population" OR "developing populations" OR "developing world" OR "less developed country" OR "less developed countries" OR "less developed nation" OR "less developed nations" OR "less developed population" OR "less developed populations" OR "less developed world" OR "lesser developed country" OR "lesser developed countries" OR "lesser developed nation" OR "lesser developed nations" OR "lesser developed population" OR "lesser developed populations" OR "lesser developed world" OR "under developed country" OR "under developed countries" OR "under developed nation" OR "under developed nations" OR "under developed population" OR "under developed populations" OR "under developed world" OR "underdeveloped country" OR "underdeveloped countries" OR "underdeveloped nation" OR "underdeveloped nations" OR "underdeveloped population" OR "underdeveloped populations" OR "underdeveloped world" OR "middle income country" OR "middle income countries" OR "middle income nation" OR "middle income nations" OR "middle income population" OR "middle income populations" OR "low income country" OR "low income countries" OR "low income nation" OR "low income nations" OR "low income population" OR "low income populations" OR "lower income country" OR "lower income countries" OR "lower income nation" OR "lower income nations" OR "lower income population" OR "lower income populations" OR "underserved country" OR "underserved countries" OR "underserved nation" OR "underserved nations" OR "underserved population" OR "underserved populations" OR "underserved world" OR "under served country" OR "under served countries" OR "under served nation" OR "under served nations" OR "under served population" OR "under served populations" OR "under served world" OR "deprived country" OR "deprived countries" OR "deprived nation" OR "deprived nations" OR "deprived population" OR "deprived populations" OR "deprived world" OR "poor country" OR "poor countries" OR "poor nation" OR "poor nations" OR "poor population" OR "poor populations" OR "poor world" OR "poorer country" OR "poorer countries" OR "poorer nation" OR "poorer nations" OR "poorer population" OR "poorer populations" OR "poorer world" OR "developing economy" OR "developing economies" OR "less developed economy" OR "less developed economies" OR "lesser developed economy" OR "lesser developed economies" OR "under developed economy" OR "under developed economies" OR "underdeveloped economy" OR "underdeveloped economies" OR "middle income economy" OR "middle income economies" OR "low income economy" OR "low income economies" OR "lower income economy" OR "lower income economies" OR "low gdp" OR "low gnp" OR "low gross domestic" OR "low gross national" OR "lower gdp" OR "lower gnp" OR "lower gross domestic" OR "lower gross national" OR lmic OR lmics OR "third world" OR "lami country" OR "lami countries" OR "transitional country" OR "transitional countries" OR "emerging economies" OR "emerging nation" OR "emerging nations"):ti,ab,kw in Trials | 101070 |
| #11 | (afghan OR afghans OR afghani OR albanian OR albanians OR algerian OR algerians OR "american samoan" OR "american samoans" OR angolan OR angolans OR antiguan OR antiguans OR barbudan OR berbudans OR argentine OR argentines OR argentinian OR argentinians OR argentinean OR argentineans OR armenian OR armenians OR aruban OR arubans OR azerbaijani OR azerbaijanis OR bahraini OR bahrainis OR bangladeshi OR bangladeshis OR bangalees OR bajan OR bajans OR belarusian OR belarusians OR byelorussian OR byelorussians OR belizean OR belizeans OR beninese OR benineses OR bhutanese OR bolivian OR bolivians OR bosnian OR bosnians OR botswana OR batswana OR brazilian OR brazilians OR brasilian OR brasilians OR bulgarian OR bulgarians OR burkinabe OR burkinese OR burundian OR burundians OR "cape verdean" OR "cape verdeans" OR "cabo verdean" OR "cabo verdeans" OR cambodian OR cambodians OR khmer OR cameroonian OR cameroonians OR "central african" OR "central africans" OR chadian OR chadians OR chilean OR chileans OR chinese OR colombian OR colombians OR comorian OR comorians OR congolese OR "costa rican" OR "costa ricans" OR ivorian OR ivorians OR croatian OR croatians OR cuban OR cubans OR cypriot OR cypriots OR czech OR czechs OR djiboutian OR djiboutians OR dominican OR dominicans OR ecuadorian OR ecuadorians OR egyptian OR egyptians OR salvadoran OR salvadorans OR "equatorial guinean" OR "equatorial guineans" OR equatoguinean OR equatoguineans OR eritrean OR eritreans OR estonian OR estonians OR swazi OR swazis OR swati OR swatis OR ethiopian OR ethiopians OR fijian OR fijians OR gabonese OR gabonaise OR gambian OR gambians OR georgian OR georgians OR ghanaian OR ghanaians OR gibraltarian OR gibraltarians OR greek OR greeks OR grenadian OR grenadians OR guamanian OR guamanians OR guatemalan OR guatemalans OR guinean OR guineans OR "bissau guinean" OR "bissau guineans" OR guyanese OR haitian OR haitians OR honduran OR hondurans OR hungarian OR hungarians OR indian OR indians OR indonesian OR indonesians OR iranian OR iranians OR iraqian OR iraqians OR iraqi OR iraqis OR manx OR jamaican OR jamaicans OR jordanian OR jordanians OR kazakhstani OR kazakhstanis OR kenyan OR kenyans OR kirabati OR kirabatian OR kirabatians OR "north korean" OR "north koreans" OR korean OR koreans OR kosovar OR kosovars OR kosovan OR kosovans OR kyrgyzstani OR kyrgyzstanis OR kyrgyz OR lao OR laotian OR laotians OR latvian OR latvians OR lebanese OR lesothan OR lesothans OR lesothonian OR lesothonians OR mosotho OR basotho OR liberian OR liberians OR libyan OR libyans OR lithuanian OR lithuanians OR macanese OR macedonian OR macedonians OR malagasy OR madagascan OR madagascans OR malawian OR malawians OR malaysian OR malaysians OR maldivian OR maldivians OR malian OR malians OR maltese OR marshallese OR marshalleses OR mauritanian OR mauritanians OR mauritian OR mauritians OR mexican OR mexicans OR micronesian OR micronesians OR moldovan OR moldovans OR mongolian OR mongolians OR mongol OR montenegrin OR montenegrins OR moroccan OR moroccans OR mozambican OR mozambicans OR burmese OR myanma OR namibian OR namibians OR nauruan OR nauruans OR nepali OR nepalese OR "netherlands antillean" OR "netherlands antilleans" OR nicaraguan OR nicaraguans OR nigerien OR nigeriens OR nigerian OR nigerians OR "northern mariana islander" OR "northern mariana islanders" OR mariana OR marianas OR omani OR omanis OR pakistani OR pakistanis OR palauan OR palauans OR panamanian OR panamanians OR "papua new guinean" OR "papua new guineans" OR paraguayan OR paraguayans OR peruvian OR peruvians OR philippine OR philippines OR philipine OR philipines OR phillipine OR phillipines OR phillippine OR phillippines OR filipino OR filipinos OR filipina OR filipinas OR polish OR pole OR poles OR portuguese OR "puerto rican" OR "puerto ricans" OR romanian OR romanians OR russian OR russians OR "soviet people" OR "soviet population" OR rwandan OR rwandans OR rwandese OR ruandan OR ruandans OR ruandese OR samoan OR samoans OR "sao tomean" OR "sao tomeans" OR santomean OR santomeans OR "saudi arabian" OR "saudi arabians" OR saudi OR saudis OR senegalese OR serbian OR serbians OR montenegrin OR montenegrins OR seychellois OR seychelloise OR seychelloises OR "sierra leonean" OR "sierra leoneans" OR slovak OR slovaks OR slovene OR slovenes OR "solomon islander" OR "solomon islanders" OR somali OR somalis OR "south african" OR "south africans" OR "south sudanese" OR "sri lankan" OR "sri lankans" OR ceylonese OR kittitian OR kittitians OR nevisian OR nevisians OR "saint lucian" OR "saint lucians" OR vincentian OR vincentians OR sudanese OR surinamese OR surinameses OR syrian OR syrians OR tajik OR tajiks OR tajikistani OR tajikistanis OR tanzanian OR tanzanians OR tanganyikan OR tanganyikans OR thai OR timorese OR timoreses OR togolese OR tongan OR tongans OR trinidadian OR trinidadians OR tobagonian OR tobagonians OR tunisian OR tunisians OR turk OR turks OR turkish OR turkmen OR turkmens OR tuvaluan OR tuvaluans OR ugandan OR ugandans OR ukrainian OR ukrainians uruguayan OR uruguayans OR uzbek OR uzbeks OR vanuatu OR vanuatuan OR vanuatuans OR venezuelan OR venezuelans OR vietnamese OR yemeni OR yemenis OR yemenite OR yemenites OR yemenese OR yugoslav OR yugoslavs OR yugoslavian OR yugoslavians OR zambian OR zambians OR zimbabwean OR zimbabweans):ti,ab,kw in Trials | 67307 |
| #12 | #10 OR #11 in Trials | 141577 |
| #13 | #3 AND #6 AND #9 AND #12 in Trials | 380 |

Key: ti = title, ab = abstract, kw = keyword, [mh XX] = medical subject heading (MeSH).

**Web of Science: Social Sciences Citation Index (SSCI) – 1900-present**

**Date of search:** October 13th 2021. **Number of retrieved references:** 516

| 6 | **#1 AND #2 AND #3 AND (#4 OR #5)** | 516 |
| --- | --- | --- |
| 5 | TS=(afghan OR afghans OR afghani OR albanian OR albanians OR algerian OR algerians OR "american samoan" OR "american samoans" OR angolan OR angolans OR antiguan OR antiguans OR barbudan OR berbudans OR argentine OR argentines OR argentinian OR argentinians OR argentinean OR argentineans OR armenian OR armenians OR aruban OR arubans OR azerbaijani OR azerbaijanis OR bahraini OR bahrainis OR bangladeshi OR bangladeshis OR bangalees OR bajan OR bajans OR belarusian OR belarusians OR byelorussian OR byelorussians OR belizean OR belizeans OR beninese OR benineses OR bhutanese OR bolivian OR bolivians OR bosnian OR bosnians OR botswana OR batswana OR brazilian OR brazilians OR brasilian OR brasilians OR bulgarian OR bulgarians OR burkinabe OR burkinese OR burundian OR burundians OR "cape verdean" OR "cape verdeans" OR "cabo verdean" OR "cabo verdeans" OR cambodian OR cambodians OR khmer OR cameroonian OR cameroonians OR "central african" OR "central africans" OR chadian OR chadians OR chilean OR chileans OR chinese OR colombian OR colombians OR comorian OR comorians OR congolese OR "costa rican" OR "costa ricans" OR ivorian OR ivorians OR croatian OR croatians OR cuban OR cubans OR cypriot OR cypriots OR czech OR czechs OR djiboutian OR djiboutians OR dominican OR dominicans OR ecuadorian OR ecuadorians OR egyptian OR egyptians OR salvadoran OR salvadorans OR "equatorial guinean" OR "equatorial guineans" OR equatoguinean OR equatoguineans OR eritrean OR eritreans OR estonian OR estonians OR swazi OR swazis OR swati OR swatis OR ethiopian OR ethiopians OR fijian OR fijians OR gabonese OR gabonaise OR gambian OR gambians OR georgian OR georgians OR ghanaian OR ghanaians OR gibraltarian OR gibraltarians OR greek OR greeks OR grenadian OR grenadians OR guamanian OR guamanians OR guatemalan OR guatemalans OR guinean OR guineans OR "bissau guinean" OR "bissau guineans" OR guyanese OR haitian OR haitians OR honduran OR hondurans OR hungarian OR hungarians OR indian OR indians OR indonesian OR indonesians OR iranian OR iranians OR iraqian OR iraqians OR iraqi OR iraqis OR manx OR jamaican OR jamaicans OR jordanian OR jordanians OR kazakhstani OR kazakhstanis OR kenyan OR kenyans OR kirabati OR kirabatian OR kirabatians OR "north korean" OR "north koreans" OR korean OR koreans OR kosovar OR kosovars OR kosovan OR kosovans OR kyrgyzstani OR kyrgyzstanis OR kyrgyz OR lao OR laotian OR laotians OR latvian OR latvians OR lebanese OR lesothan OR lesothans OR lesothonian OR lesothonians OR mosotho OR basotho OR liberian OR liberians OR libyan OR libyans OR lithuanian OR lithuanians OR macanese OR macedonian OR macedonians OR malagasy OR madagascan OR madagascans OR malawian OR malawians OR malaysian OR malaysians OR maldivian OR maldivians OR malian OR malians OR maltese OR marshallese OR marshalleses OR mauritanian OR mauritanians OR mauritian OR mauritians OR mexican OR mexicans OR micronesian OR micronesians OR moldovan OR moldovans OR mongolian OR mongolians OR mongol OR montenegrin OR montenegrins OR moroccan OR moroccans OR mozambican OR mozambicans OR burmese OR myanma OR namibian OR namibians OR nauruan OR nauruans OR nepali OR nepalese OR "netherlands antillean" OR "netherlands antilleans" OR nicaraguan OR nicaraguans OR nigerien OR nigeriens OR nigerian OR nigerians OR "northern mariana islander" OR "northern mariana islanders" OR mariana OR marianas OR omani OR omanis OR pakistani OR pakistanis OR palauan OR palauans OR panamanian OR panamanians OR "papua new guinean" OR "papua new guineans" OR paraguayan OR paraguayans OR peruvian OR peruvians OR philippine OR philippines OR philipine OR philipines OR phillipine OR phillipines OR phillippine OR phillippines OR filipino OR filipinos OR filipina OR filipinas OR polish OR pole OR poles OR portuguese OR "puerto rican" OR "puerto ricans" OR romanian OR romanians OR russian OR russians OR "soviet people" OR "soviet population" OR rwandan OR rwandans OR rwandese OR ruandan OR ruandans OR ruandese OR samoan OR samoans OR "sao tomean" OR "sao tomeans" OR santomean OR santomeans OR "saudi arabian" OR "saudi arabians" OR saudi OR saudis OR senegalese OR serbian OR serbians OR montenegrin OR montenegrins OR seychellois OR seychelloise OR seychelloises OR "sierra leonean" OR "sierra leoneans" OR slovak OR slovaks OR slovene OR slovenes OR "solomon islander" OR "solomon islanders" OR somali OR somalis OR "south african" OR "south africans" OR "south sudanese" OR "sri lankan" OR "sri lankans" OR ceylonese OR kittitian OR kittitians OR nevisian OR nevisians OR "saint lucian" OR "saint lucians" OR vincentian OR vincentians OR sudanese OR surinamese OR surinameses OR syrian OR syrians OR tajik OR tajiks OR tajikistani OR tajikistanis OR tanzanian OR tanzanians OR tanganyikan OR tanganyikans OR thai OR timorese OR timoreses OR togolese OR tongan OR tongans OR trinidadian OR trinidadians OR tobagonian OR tobagonians OR tunisian OR tunisians OR turk OR turks OR turkish OR turkmen OR turkmens OR tuvaluan OR tuvaluans OR ugandan OR ugandans OR ukrainian OR "ukrainians uruguayan" OR uruguayans OR uzbek OR uzbeks OR vanuatu OR vanuatuan OR vanuatuans OR venezuelan OR venezuelans OR vietnamese OR yemeni OR yemenis OR yemenite OR yemenites OR yemenese OR yugoslav OR yugoslavs OR yugoslavian OR yugoslavians OR zambian OR zambians OR zimbabwean OR zimbabweans) | 533 748 |
| 4 | TS=(afghanistan OR albania OR algeria OR "american samoa" OR angola OR "antigua and barbuda" OR antigua OR barbuda OR argentina OR armenia OR armenian OR aruba OR azerbaijan OR bahrain OR bangladesh OR barbados OR "republic of belarus" OR belarus OR byelarus OR belorussia OR byelorussian OR belize OR "british honduras" OR benin OR dahomey OR bhutan OR bolivia OR "bosnia and herzegovina" OR bosnia OR herzegovina OR botswana OR bechuanaland OR brazil OR brasil OR bulgaria OR "burkina faso" OR "burkina fasso" OR "upper volta" OR burundi OR urundi OR "cabo verde" OR "cape verde" OR cambodia OR kampuchea OR "khmer republic" OR cameroon OR cameron OR cameroun OR "central african republic" OR "ubangi shari" OR chad OR chile OR china OR colombia OR comoros OR "comoro islands" OR "iles comores" OR mayotte OR "democratic republic of the congo" OR "democratic republic congo" OR congo OR zaire OR "costa rica" OR "cote d’ivoire" OR "cote d’ ivoire" OR "cote divoire" OR "cote d ivoire" OR "ivory coast" OR croatia OR cuba OR cyprus OR "czech republic" OR czechoslovakia OR djibouti OR "french somaliland" OR dominica OR "dominican republic" OR ecuador OR egypt OR "united arab republic" OR "el salvador" OR "equatorial guinea" OR "spanish guinea" OR eritrea OR estonia OR eswatini OR swaziland OR ethiopia OR fiji OR gabon OR "gabonese republic" OR gambia OR "georgia (republic)" OR georgian OR ghana OR "gold coast" OR gibraltar OR greece OR grenada OR guam OR guatemala OR guinea OR "guinea bissau" OR guyana OR "british guiana" OR haiti OR hispaniola OR honduras OR hungary OR india OR indonesia OR timor OR iran OR iraq OR "isle of man" OR jamaica OR jordan OR kazakhstan OR kazakh OR kenya OR "democratic people’s republic of korea" OR "republic of korea" OR "north korea" OR "south korea" OR korea OR kosovo OR kyrgyzstan OR kirghizia OR kirgizstan OR "kyrgyz republic" OR kirghiz OR laos OR "lao pdr" OR "lao people's democratic republic" OR latvia OR lebanon OR "lebanese republic" OR lesotho OR basutoland OR liberia OR libya OR "libyan arab jamahiriya" OR lithuania OR macau OR macao OR "republic of north macedonia" OR macedonia OR madagascar OR "malagasy republic" OR malawi OR nyasaland OR malaysia OR "malay federation" OR "malaya federation" OR maldives OR "indian ocean islands" OR "indian ocean" OR mali OR malta OR micronesia OR "federated states of micronesia" OR kiribati OR "marshall islands" OR nauru OR "northern mariana islands" OR palau OR tuvalu OR mauritania OR mauritius OR mexico OR moldova OR moldovian OR mongolia OR montenegro OR morocco OR ifni OR mozambique OR "portuguese east africa" OR myanmar OR burma OR namibia OR nepal OR "netherlands antilles" OR nicaragua OR niger OR nigeria OR oman OR muscat OR pakistan OR panama OR "papua new guinea" OR "new guinea" OR paraguay OR peru OR philippines OR philipines OR phillipines OR phillippines OR poland OR "polish people's republic" OR portugal OR "portuguese republic" OR "puerto rico" OR romania OR russia OR "russian federation" OR ussr OR "soviet union" OR "union of soviet socialist republics" OR rwanda OR ruanda OR samoa OR "pacific islands" OR polynesia OR "samoan islands" OR "navigator island" OR "navigator islands" OR "sao tome and principe" OR "saudi arabia" OR senegal OR serbia OR seychelles OR "sierra leone" OR slovakia OR "slovak republic" OR slovenia OR melanesia OR "solomon island" OR "solomon islands" OR "norfolk island" OR "norfolk islands" OR somalia OR "south africa" OR "south sudan" OR "sri lanka" OR ceylon OR "saint kitts and nevis" OR "st. kitts and nevis" OR "saint lucia" OR "st. lucia" OR "saint vincent and the grenadines" OR "saint vincent" OR "st. vincent" OR grenadines OR sudan OR suriname OR surinam OR "dutch guiana" OR "netherlands guiana" OR syria OR "syrian arab republic" OR tajikistan OR tadjikistan OR tadzhikistan OR tadzhik OR tanzania OR tanganyika OR thailand OR siam OR "timor leste" OR "east timor" OR togo OR "togolese republic" OR tonga OR "trinidad and tobago" OR trinidad OR tobago OR tunisia OR turkey OR turkmenistan OR turkmen OR uganda OR ukraine OR uruguay OR uzbekistan OR uzbek OR vanuatu OR "new hebrides" OR venezuela OR vietnam OR "viet nam" OR "middle east" OR "west bank" OR gaza OR palestine OR yemen OR yugoslavia OR zambia OR zimbabwe OR "northern rhodesia" OR "global south" OR "africa south of the sahara" OR "sub-saharan africa" OR "subsaharan africa" OR "africa, central" OR "central africa" OR "africa, northern" OR "north africa" OR "northern africa" OR magreb OR maghrib OR sahara OR "africa, southern" OR "southern africa" OR "africa, eastern" OR "east africa" OR "eastern africa" OR "africa, western" OR "west africa" OR "western africa" OR "west indies" OR "indian ocean islands" OR caribbean OR "central america" OR "latin america" OR "south and central america" OR "south america" OR "asia, central" OR "central asia" OR "asia, northern" OR "north asia" OR "northern asia" OR "asia, southeastern" OR "southeastern asia" OR "south eastern asia" OR "southeast asia" OR "south east asia" OR "asia, western" OR "western asia" OR "europe, eastern" OR "east europe" OR "eastern europe" OR "developing country" OR "developing countries" OR "developing nation$" OR "developing population$" OR "developing world" OR "less developed countr*" OR "less developed nation$" OR "less developed population$" OR "less developed world" OR "lesser developed countr*" OR "lesser developed nation$" OR "lesser developed population$" OR "lesser developed world" OR "under developed countr*" OR "under developed nation$" OR "under developed population$" OR "under developed world" OR "underdeveloped countr*" OR "underdeveloped nation$" OR "underdeveloped population$" OR "underdeveloped world" OR "middle income countr*" OR "middle income nation$" OR "middle income population$" OR "low income countr*" OR "low income nation$" OR "low income population$" OR "lower income countr*" OR "lower income nation$" OR "lower income population$" OR "underserved countr*" OR "underserved nation$" OR "underserved population$" OR "underserved world" OR "under served countr*" OR "under served nation$" OR "under served population$" OR "under served world" OR "deprived countr*" OR "deprived nation$" OR "deprived population$" OR "deprived world" OR "poor countr*" OR "poor nation$" OR "poor population$" OR "poor world" OR "poorer countr*" OR "poorer nation$" OR "poorer population$" OR "poorer world" OR "developing econom*" OR "less developed econom*" OR "lesser developed econom*" OR "under developed econom*" OR "underdeveloped econom*" OR "middle income econom*" OR "low income econom*" OR "lower income econom*" OR "low gdp" OR "low gnp" OR "low gross domestic" OR "low gross national" OR "lower gdp" OR "lower gnp" OR "lower gross domestic" OR "lower gross national" OR lmic OR lmics OR "third world" OR "lami countr*" OR "transitional countr*" OR "emerging economies" OR "emerging nation$") | 1 033 507 |
| 3 | TS=(nursery OR nurseries OR kindergarten$ OR kindergarden$ OR pre-school$ OR preschool$ OR school$ OR "junior high$" OR high-school$ OR elementary-school$ OR junior-high$ OR middle-school$ OR class OR classes OR classroom$ OR pupil$ OR teacher$) | 636 017 |
| 2 | TS=(implement* OR barrier* OR facilitator* OR determinant$) | 561 288 |
| 1 | TS=(((nutrition OR diet OR dietary OR food OR obesity OR overweight OR "physical* activ*" OR "physical* inactiv*" OR sport$ OR exercise$ OR sedentary OR "active travel" OR "active transport*" OR "active commut*" OR "transport mode" OR "transportation mode" OR "travel mode" OR cycle OR cycling OR bicycle OR bicycling OR cyclist* OR walking OR walk*) NEAR/3 (intervention$ OR program* OR policies OR policy OR promotion OR campaign$)) OR "double-duty action$") | 47 593 |

Key: TS = topic, which includes title, abstract, author keywords and Web of Science Keywords Plus, NEAR/n = word distance of maximum n words

**Scopus (Elsevier)**

**Date of search:** October 13th 2021. **Number of retrieved references:** 1621

| 6 | #1 AND #2 AND #3 AND (#4 OR #5) | 1621 |
| --- | --- | --- |
| 5 | TITLE-ABS-KEY ( ( afghan  OR  afghans  OR  afghani  OR  "albanian* algerian*"  OR  "american samoan*"  OR  angolan*  OR  antiguan*  OR  barbudan*  OR  argentine*  OR  argentinian*  OR  argentinean*  OR  armenian*  OR  aruban*  OR  azerbaijani*  OR  bahraini*  OR  bangladeshi*  OR  bangalees  OR  bajan*  OR  belarusian*  OR  byelorussian*  OR  belizean*  OR  beninese*  OR  bhutanese  OR  bolivian*  OR  bosnian*  OR  botswana  OR  batswana  OR  brazilian*  OR  brasilian*  OR  bulgarian*  OR  burkinabe  OR  burkinese  OR  burundian*  OR  "cape verdean*"  OR  "cabo verdean*"  OR  cambodian*  OR  khmer  OR  cameroonian*  OR  "central african*"  OR  chadian*  OR  chilean*  OR  chinese  OR  colombian*  OR  comorian*  OR  congolese  OR  "costa rican*"  OR  ivorian*  OR  croatian*  OR  cuban*  OR  cypriot*  OR  czech*  OR  djiboutian*  OR  dominican*  OR  ecuadorian*  OR  egyptian*  OR  salvadoran*  OR  "equatorial guinean*"  OR  equatoguinean*  OR  eritrean*  OR  estonian*  OR  swazi*  OR  swati*  OR  ethiopian*  OR  fijian  OR  gabonese  OR  gabonaise  OR  gambian*  OR  georgian*  OR  ghanaian*  OR  gibraltarian*  OR  greek*  OR  grenadian*  OR  guamanian*  OR  guatemalan*  OR  guinean*  OR  "bissau guinean*"  OR  guyanese  OR  haitian*  OR  honduran*  OR  hungarian*  OR  indian*  OR  indonesian*  OR  iranian*  OR  iraqian*  OR  iraqi*  OR  manx  OR  jamaican*  OR  jordanian*  OR  kazakhstani*  OR  kenyan*  OR  kirabati  OR  kirabatian*  OR  "north korean*"  OR  korean*  OR  kosovar*  OR  kosovan*  OR  kyrgyz*  OR  lao  OR  laotian*  OR  latvian*  OR  lebanese  OR  lesothan*  OR  lesothonian*  OR  mosotho  OR  basotho  OR  liberian*  OR  libyan*  OR  lithuanian*  OR  macanese  OR  macedonian*  OR  malagasy  OR  madagascan*  OR  malawian*  OR  malaysian*  OR  maldivian*  OR  malian*  OR  maltese  OR  marshallese*  OR  mauritanian*  OR  mauritian*  OR  mexican*  OR  micronesian*  OR  moldovan*  OR  mongolian*  OR  mongol  OR  montenegrin*  OR  moroccan*  OR  mozambican*  OR  burmese  OR  myanma  OR  namibian*  OR  nauruan*  OR  nepali  OR  nepalese  OR  "netherlands antillean*"  OR  nicaraguan*  OR  nigerien*  OR  nigerian*  OR  "northern mariana islander*"  OR  mariana*  OR  omani*  OR  pakistani*  OR  palauan*  OR  panamanian*  OR  "papua new guinean*"  OR  paraguayan*  OR  peruvian*  OR  philippine*  OR  philipine*  OR  phillipine*  OR  phillippine*  OR  filipino*  OR  filipina*  OR  polish  OR  pole  OR  poles  OR  portuguese  OR  "puerto rican*"  OR  romanian*  OR  russian*  OR  "soviet people"  OR  "soviet population"  OR  rwandan*  OR  rwandese  OR  ruandan*  OR  ruandese  OR  samoan*  OR  "sao tomean*"  OR  santomean*  OR  "saudi arabian*"  OR  saudi*  OR  senegalese  OR  serbian*  OR  montenegrin*  OR  seychellois  OR  seychelloise*  OR  "sierra leonean*"  OR  slovak*  OR  slovene*  OR  "solomon islander*"  OR  somali*  OR  "south african*"  OR  "south sudanese"  OR  "sri lankan*"  OR  ceylonese  OR  kittitian*  OR  nevisian*  OR  "saint lucian*"  OR  vincentian*  OR  sudanese  OR  surinamese*  OR  syrian*  OR  tajik*  OR  tajikistani*  OR  tanzanian*  OR  tanganyikan*  OR  thai  OR  timorese*  OR  togolese  OR  tongan*  OR  trinidadian*  OR  tobagonian*  OR  tunisian*  OR  turk*  OR  turkish  OR  turkmen*  OR  tuvaluan*  OR  ugandan*  OR  ukrainian*  OR  uruguayan*  OR  uzbek*  OR  vanuatu*  OR  venezuelan*  OR  vietnamese  OR  yemeni*  OR  yemenite*  OR  yemenese  OR  yugoslav*  OR  yugoslavian*  OR  zambian*  OR  zimbabwean* ) ) | 3 361 607 |
| 4 | TITLE-ABS-KEY ( ( afghanistan  OR  albania  OR  algeria  OR  "american samoa"  OR  angola  OR  "antigua and barbuda"  OR  antigua  OR  barbuda  OR  argentina  OR  armenia  OR  armenian  OR  aruba  OR  azerbaijan  OR  bahrain  OR  bangladesh  OR  barbados  OR  "republic of belarus"  OR  belarus  OR  byelarus  OR  belorussia  OR  byelorussian  OR  belize  OR  "british honduras"  OR  benin  OR  dahomey  OR  bhutan  OR  bolivia  OR  "bosnia and herzegovina"  OR  bosnia  OR  herzegovina  OR  botswana  OR  bechuanaland  OR  brazil  OR  brasil  OR  bulgaria  OR  "burkina faso"  OR  "burkina fasso"  OR  "upper volta"  OR  burundi  OR  urundi  OR  "cabo verde"  OR  "cape verde"  OR  cambodia  OR  kampuchea  OR  "khmer republic"  OR  cameroon  OR  cameron  OR  cameroun  OR  "central african republic"  OR  "ubangi shari"  OR  chad  OR  chile  OR  china  OR  colombia  OR  comoros  OR  "comoro islands"  OR  "iles comores"  OR  mayotte  OR  "democratic republic of the congo"  OR  "democratic republic congo"  OR  congo  OR  zaire  OR  "costa rica"  OR  "cote d'ivoire"  OR  "cote d' ivoire"  OR  "cote divoire"  OR  "cote d ivoire"  OR  "ivory coast"  OR  croatia  OR  cuba  OR  cyprus  OR  "czech republic"  OR  czechoslovakia  OR  djibouti  OR  "french somaliland"  OR  dominica  OR  "dominican republic"  OR  ecuador  OR  egypt  OR  "united arab republic"  OR  "el salvador"  OR  "equatorial guinea"  OR  "spanish guinea"  OR  eritrea  OR  estonia  OR  eswatini  OR  swaziland  OR  ethiopia  OR  fiji  OR  gabon  OR  "gabonese republic"  OR  gambia  OR  "georgia (republic)"  OR  georgian  OR  ghana  OR  "gold coast"  OR  gibraltar  OR  greece  OR  grenada  OR  guam  OR  guatemala  OR  guinea  OR  "guinea bissau"  OR  guyana  OR  "british guiana"  OR  haiti  OR  hispaniola  OR  honduras  OR  hungary  OR  india  OR  indonesia  OR  timor  OR  iran  OR  iraq  OR  "isle of man"  OR  jamaica  OR  jordan  OR  kazakhstan  OR  kazakh  OR  kenya  OR  "democratic people's republic of korea"  OR  "republic of korea"  OR  "north korea"  OR  "south korea"  OR  korea  OR  kosovo  OR  kyrgyzstan  OR  kirghizia  OR  kirgizstan  OR  "kyrgyz republic"  OR  kirghiz  OR  laos  OR  "lao pdr"  OR  "lao people's democratic republic"  OR  latvia  OR  lebanon  OR  "lebanese republic"  OR  lesotho  OR  basutoland  OR  liberia  OR  libya  OR  "libyan arab jamahiriya"  OR  lithuania  OR  macau  OR  macao  OR  "republic of north macedonia"  OR  macedonia  OR  madagascar  OR  "malagasy republic"  OR  malawi  OR  nyasaland  OR  malaysia  OR  "malay federation"  OR  "malaya federation"  OR  maldives  OR  "indian ocean islands"  OR  "indian ocean"  OR  mali  OR  malta  OR  micronesia  OR  "federated states of micronesia"  OR  kiribati  OR  "marshall islands"  OR  nauru  OR  "northern mariana islands"  OR  palau  OR  tuvalu  OR  mauritania  OR  mauritius  OR  mexico  OR  moldova  OR  moldovian  OR  mongolia  OR  montenegro  OR  morocco  OR  ifni  OR  mozambique  OR  "portuguese east africa"  OR  myanmar  OR  burma  OR  namibia  OR  nepal  OR  "netherlands antilles"  OR  nicaragua  OR  niger  OR  nigeria  OR  oman  OR  muscat  OR  pakistan  OR  panama  OR  "papua new guinea"  OR  "new guinea"  OR  paraguay  OR  peru  OR  philippines  OR  philipines  OR  phillipines  OR  phillippines  OR  poland  OR  "polish people's republic"  OR  portugal  OR  "portuguese republic"  OR  "puerto rico"  OR  romania  OR  russia  OR  "russian federation"  OR  ussr  OR  "soviet union"  OR  "union of soviet socialist republics"  OR  rwanda  OR  ruanda  OR  samoa  OR  "pacific islands"  OR  polynesia  OR  "samoan islands"  OR  "navigator island"  OR  "navigator islands"  OR  "sao tome and principe"  OR  "saudi arabia"  OR  senegal  OR  serbia  OR  seychelles  OR  "sierra leone"  OR  slovakia  OR  "slovak republic"  OR  slovenia  OR  melanesia  OR  "solomon island"  OR  "solomon islands"  OR  "norfolk island"  OR  "norfolk islands"  OR  somalia  OR  "south africa"  OR  "south sudan"  OR  "sri lanka"  OR  ceylon  OR  "saint kitts and nevis"  OR  "st. kitts and nevis"  OR  "saint lucia"  OR  "st. lucia"  OR  "saint vincent and the grenadines"  OR  "saint vincent"  OR  "st. vincent"  OR  grenadines  OR  sudan  OR  suriname  OR  surinam  OR  "dutch guiana"  OR  "netherlands guiana"  OR  syria  OR  "syrian arab republic"  OR  tajikistan  OR  tadjikistan  OR  tadzhikistan  OR  tadzhik  OR  tanzania  OR  tanganyika  OR  thailand  OR  siam  OR  "timor leste"  OR  "east timor"  OR  togo  OR  "togolese republic"  OR  tonga  OR  "trinidad and tobago"  OR  trinidad  OR  tobago  OR  tunisia  OR  turkey  OR  turkmenistan  OR  turkmen  OR  uganda  OR  ukraine  OR  uruguay  OR  uzbekistan  OR  uzbek  OR  vanuatu  OR  "new hebrides"  OR  venezuela  OR  vietnam  OR  "viet nam"  OR  "middle east"  OR  "west bank"  OR  gaza  OR  palestine  OR  yemen  OR  yugoslavia  OR  zambia  OR  zimbabwe  OR  "northern rhodesia"  OR  "global south"  OR  "africa south of the sahara"  OR  "sub-saharan africa"  OR  "subsaharan africa"  OR  "africa, central"  OR  "central africa"  OR  "africa, northern"  OR  "north africa"  OR  "northern africa"  OR  magreb  OR  maghrib  OR  sahara  OR  "africa, southern"  OR  "southern africa"  OR  "africa, eastern"  OR  "east africa"  OR  "eastern africa"  OR  "africa, western"  OR  "west africa"  OR  "western africa"  OR  "west indies"  OR  "indian ocean islands"  OR  caribbean  OR  "central america"  OR  "latin america"  OR  "south and central america"  OR  "south america"  OR  "asia, central"  OR  "central asia"  OR  "asia, northern"  OR  "north asia"  OR  "northern asia"  OR  "asia, southeastern"  OR  "southeastern asia"  OR  "south eastern asia"  OR  "southeast asia"  OR  "south east asia"  OR  "asia, western"  OR  "western asia"  OR  "europe, eastern"  OR  "east europe"  OR  "eastern europe"  OR  "developing country"  OR  "developing countries"  OR  "developing nation*"  OR  "developing population*"  OR  "developing world"  OR  "less developed countr*"  OR  "less developed nation*"  OR  "less developed population*"  OR  "less developed world"  OR  "lesser developed countr*"  OR  "lesser developed nation*"  OR  "lesser developed population*"  OR  "lesser developed world"  OR  "under developed countr*"  OR  "under developed nation*"  OR  "under developed population*"  OR  "under developed world"  OR  "underdeveloped countr*"  OR  "underdeveloped nation*"  OR  "underdeveloped population*"  OR  "underdeveloped world"  OR  "middle income countr*"  OR  "middle income nation*"  OR  "middle income population*"  OR  "low income countr*"  OR  "low income nation*"  OR  "low income population*"  OR  "lower income countr*"  OR  "lower income nation*"  OR  "lower income population*"  OR  "underserved countr*"  OR  "underserved nation*"  OR  "underserved population*"  OR  "underserved world"  OR  "under served countr*"  OR  "under served nation*"  OR  "under served population*"  OR  "under served world"  OR  "deprived countr*"  OR  "deprived nation*"  OR  "deprived population*"  OR  "deprived world"  OR  "poor countr*"  OR  "poor nation*"  OR  "poor population*"  OR  "poor world"  OR  "poorer countr*"  OR  "poorer nation*"  OR  "poorer population*"  OR  "poorer world"  OR  "developing econom*"  OR  "less developed econom*"  OR  "lesser developed econom*"  OR  "under developed econom*"  OR  "underdeveloped econom*"  OR  "middle income econom*"  OR  "low income econom*"  OR  "lower income econom*"  OR  "low gdp"  OR  "low gnp"  OR  "low gross domestic"  OR  "low gross national"  OR  "lower gdp"  OR  "lower gnp"  OR  "lower gross domestic"  OR  "lower gross national"  OR  lmic  OR  lmics  OR  "third world"  OR  "lami countr*"  OR  "transitional countr*"  OR  "emerging economies"  OR  "emerging nation*" ) ) | 6 150 717 |
| 3 | TITLE-ABS-KEY ( nursery  OR  nurseries  OR  kindergarten*  OR  kindergarden*  OR  pre-school*  OR  preschool*  OR  school*  OR  "junior high*"  OR  high-school*  OR  elementary-school*  OR  junior-high*  OR  middle-school*  OR  class  OR  classes  OR  classroom*  OR  pupil*  OR  teacher* ) | 4 316 448 |
| 2 | TITLE-ABS-KEY((implement* OR barrier* OR facilitator* OR determinant*)) | 4 166 348 |
| 1 | TITLE-ABS-KEY ( ( ( ( nutrition  OR  diet  OR  dietary  OR  food  OR  obesity  OR  overweight  OR  "physical* activ*"  OR  "physical* inactiv*"  OR  sport*  OR  exercise*  OR  sedentary  OR  "active travel"  OR  "active transport*"  OR  "active commut*"  OR  "transport mode"  OR  "transportation mode"  OR  "travel mode"  OR  cycle  OR  cycling  OR  bicycle  OR  bicycling  OR  cyclist*  OR  walking  OR  walk* )  W/3  ( intervention*  OR  program*  OR  policies  OR  policy  OR  promotion  OR  campaign* ) )  OR  "double-duty action*" ) ) | 149 707 |

Key: TITLE-ABS-KEY = TITLE = title, ABS = abstract, KEY = a combined field that searches author supplied keywords, EMTREE subject headings, other keywords, trade names and chemical names , W/n = word distance of maximum n words, , {} = wildcard for exact searching

**Global Index Medicus (WHO)**
Advanced search

**Date of search:** October 18th 2021. **Number of retrieved references:** 1288.
**After duplicates removed**: 1277

(tw:((((nutrition or diet or dietary or food or obesity or overweight or physical* activ* or physical* inactiv* or sport* or exercise* or sedentary or "active travel" or "active transport*" or "active commut*" or "transport mode" or "transportation mode" or "travel mode" or cycle or cycling or bicycle or bicycling or cyclist* or walking or walk*) AND (intervention* or program* or policies or policy or promotion or campaign*)) or double-duty action*))) AND (tw:((implement* OR barrier* OR facilitator* OR determinant*))) AND (tw:((nursery OR nurseries OR kindergarten* OR kindergarden* OR pre-school* OR preschool* OR school* OR "junior high*" OR high-school* OR elementary-school* OR junior-high* OR middle-school* OR class OR classes OR classroom* OR pupil* OR teacher*)))

TW= title, abstract, subject.
