# Supplementary material for: Barriers and facilitators to implementation of nutrition-related actions in school settings in low- and middle-income countries (LMICs): a qualitative systematic review using the Consolidated Framework for Implementation Research (CFIR)
Source: Implement Sci Commun. 2023 Jun 27;4:73. doi: 10.1186/s43058-023-00454-y (PMC10294384; doi:10.1186/s43058-023-00454-y)
Supplement: Supplementary file 3 — Additional file 3. Summary of barriers and facilitators. [file 43058_2023_454_MOESM3_ESM.docx]

Implementation of Double Duty Actions in Low and Middle Income Countries_Systematic_Review

Nodes

| Name | Description | Papers | Barriers | Facilitators |
| --- | --- | --- | --- | --- |
| CFIR Constructs |  |  |  |  |
| I. INNOVATION CHARACTERISTICS | Characteristics of the intervention being implemented into a particular organization. Ideally, we'd have a definition of the "essential core" components of the intervention versus the "flexible periphery." |  |  |  |
| A. Innovation Source | Perception of key stakeholders about whether the intervention is externally or internally developed. | 2 | - Difficult to adopt an external program and integrate in existing school schedules (Chavez, 2020) - Significant doubts about the program, when teachers were not appointment leaders (when run from outside the school setting) (Iddrisu, 2017) |  |
| B. Evidence Strength & Quality | Stakeholders’ perceptions of the quality and validity of evidence supporting the belief that the intervention will have desired outcomes. | 2 | - Private sector doubts about the evidence linking SSB and childhood obesity (Moise, 2011) - Politicians requesting stronger evidence, and evidence particular to the country (Philippines) on the links between SSB and obesity (Reeve, 2018) |  |
| C. Relative Advantage | Stakeholders’ perception of the advantage of implementing the intervention versus an alternative solution. |  |  |  |
| D. Adaptability | The degree to which an intervention can be adapted, tailored, refined, or reinvented to meet local needs. | 4 |  | - Menus can be adapted, as some ingredients are sometimes missing (Cervato Mancuso, 2013) - Concessionaire could choose between home made food, and packaged food, preferring the former, as the profit margin was greater (Jensen, 2021) - Menus are adapted accordingly when ingredients are missing (Shrestha, 2020) - Menus were left up to the particular village and school community to design based on seasonal foods (Studdert, 2004) |
| E. Trialability | The ability to test the intervention on a small scale in the organization [8], and to be able to reverse course (undo implementation) if warranted. |  |  |  |
| F. Complexity | Perceived difficulty of implementation, reflected by duration, scope, radicalness, disruptiveness, centrality, and intricacy and number of steps required to implement | 11 | - Bureaucratic rules for filling in reimbursement forms for cooperatives were too difficult to understand, resulted in delays in payment (Beesley, 2013) - Some requirements, such that each cooperative serve 5000 schools, or each cooperative is made up of 10 members were not explained (Beesley, 2013) - Rules for management of cooperatives (such that new members could not be added, old members could not be cancelled, as well as that each had to come from different wards, where travel was difficult, so they could not meet, nor did they have the office space to meet) were difficult to implement (Beesley, 2013) - So students from different classrooms and school years would participate and would not loose class, when in sessions of the program, sessions were organized between morning and afternoon shifts, which significantly lowered the participation of students (Chavez, 2020) - The complexity in having different program parts such as focus on mental health and obesity related programs as part of Health Promoting Schools initiative, made it difficult to integrate everything (Chavez, 2020) - Excess bureaucracy for participation of family farmers (de Fatima Vilela, 2019) - Low participation of family farmers due to bureaucracy (Ferreira, 2019) - Requirements to have special certificates lowered the participation of family farmers (Ferreira, 2019) - Frequent shifting of the program from one ministry to another make it difficult to operate and monitor (Iddrisu, 2017) - Bureaucratic procedures caused late payments to service providers (Mafugu, 2021) - More learners in the school than budgeted for, which made quantities of food insufficient, due to inability to register students enrolled 10 days after school starts (Mafugu, 2021) - Although schools may enrol more students than the previous year, Education does not make the adjustments in funding (Mawela, 2018) - Complex legal frameworks and policy making environment, together with lobbying by private sector make it very difficult to implement any restrictions on SSB (Moise, 2011) - Bureaucracy in regard to inclusion of family farmers in the school meal program (Mossmann, 2017) - Rules required some standardization of production by farmers, which farmers were not very willing to comply with (Mossmann, 2017) - Public procurement calls not specific enough in regard to some points (types of food, quantities, delivery schedule) which then caused implementation challenges (Mossmann, 2017 - Difficulty involving family farmers due to institutional difficulties and infrastructure and logistic problems (Scwartzman, 2017) - Project leaders had to obtain approval for project and access to schools through government bodies which was a complex process. After the approval and access, school principal and teachers would have to be convinced to participate as well (Skar, 2015) |  |
| G. Design Quality & Packaging | Perceived excellence in how the intervention is bundled, presented, and assembled | 20 | - Food tasted sour, giving the impression to children that all healthy food is sour and without any salt which they did not like (Azizan, 2021) - Repetition of same menu was not appreciated (rice and sausage every day) (Cervato Mancuso, 2013) - Food waste (Cervato Mancuso, 2013) - Lack of diversity of products (de Fatima Vilela, 2019) - Only one meal cooked, and no provisions for those with dietary concerns (ex. Pupil could not eat beans due to stomach pains) (Essuman, 2013) - Food not appealing (Essuman, 2013) - Correct use of meal planning tool made meals too expensive. Meals that satisfied the nutritional requirements based on use of the meal planning tool were more expensive than the per meal allowance (Fernandes, 2016) - Quantity and variety supplied by family farmers is not sufficient (Ferreira, 2019) - No quality criteria on ingredients used in home made products (Girona, 2018) - Insufficient quantity of food planned and delivered (Mafugu, 2021) - School specific food preferences not taken into consideration when providing food (ex. Culture, allergies, sickness) (Mafugu, 2021) - Provision of lunch only and not breakfast, or provision of breakfast only to primary schools and not secondary (Mawela, 2018) - Quantity of food insufficient (Mohanty, 2014) - Low quality food sometimes provided (rotten eggs, rice and pulses of low quality) (Mohanty, 2014) - More interactive activities, greater variety of activities should be part of the program (ex. Scavenger hunt, workshops) (Moraes, 2018) - Farmers did not provide necessary quantities and diversity of products (Mossmann, 2017) - No fruit and vegetables served (Roothaert, 2021) - Concerns about quality and safety of products (ex. Use of agro-chemicals) (Roothaert, 2021) - Meal portions not sufficient (Roothaert, 2021) - Meal program lack quality criteria (Skar, 2015) - Quantity of food not sufficient (Sulemana, 2013) | - Menus becoming more ‘fresh’ rather than ‘fat’ and full of chemicals (Cervato Mancuso, 2013) - Making sure only organic fertilizers are used, and the hygienic conditions in the kitchen are good (Cervato Mancuso, 2013) - Menu planning tool was easy to use and provided useful knowledge (Fernandes, 2016) - Provision of measurement tools to use with the meal planner was useful (Fernandes, 2016) - Emphasis on home made products (Girona, 2018) - Program content on health damages caused by sugary drinks and processed foods changed teacher purchasing practices (Merida Rios, 2019) - Videos and practical activities as a more acceptable tools of conveying knowledge than talks (Merida Rios, 2019) - Food of good quality as an incentive to come to school (Mohanty, 2014) - Fresh local food of good quality (Rocha, 2018) - Menus are standardized and the cooking is based on the menus (Shrestha, 2020) - Health education sessions for children identified as ‘cornerstone’ of various interventions (Skar, 2015) - School feeding program snacks were diverse (Studdert, 2004) - School feeding program snacks were of good nutritional value (Studdert, 2004) - Use of local products (Studdert, 2004) - Video clippings are a good way to convey knowledge on health to students (Yuvaraj, 2019) |
| H. Cost | Costs of the intervention and costs associated with implementing that intervention including investment, supply, and opportunity costs. | 12 | - Healthy food is perceived as expensive (Azizan, 2021) - Funds allocated to the program were not adjusted for inflation (Beesley, 2013) - High cost of fruit and vegetable is perceived as a barrier (Fernandes, 2016) - Funds are not enough for food that would satisfy the nutritional requirements (Ferreira, 2019) - Home made products perceived as more expensive than packaged (Girona, 2018) - The cost of products from family farmers is higher than products provided from larger producers (Mossmann, 2017) - When children had to purchase food at school, highly processed foods were cheaper than locally prepared alternatives (Reeve, 2018) - For children and families, affordability of healthy foods and beverages compared to unhealthy alternatives was a key barrier to implementation of the Standards (Reeve, 2021) - Funds not sufficient to implement all components of a health and nutrition program (Shrestha, 2019) - Short shelf life and spoilage of healthy foods increases their cost (Skar, 2015) - Economic crisis caused an increase in price of food, which had some influence on snack size and quality, but not frequency of delivery (Studdert, 2004) - The amount allocated per child as part of the school feeding program was not sufficient (Sulemana, 2013) | - When farmers, in addition to the government made some investment in production, it was possible to deliver the necessary products and quantities linked to the school meal program (Mossmann, 2017) |
| II. OUTER SETTING | Generally, the outer setting includes the economic, political, and social context within which an organization resides. Changes in the outer setting can influence implementation, often mediated through changes in the inner setting. |  |  |  |
| A. Needs & Resources of Those Served by the Organization | The extent to which patient needs, as well as barriers and facilitators to meet those needs are accurately known and prioritized by the organization. | 13 | - When school food is sold and not provided for free, the high cost of healthy food is a concern as principals prefer to sell cheap food that does not satisfy the nutritional Guidelines, due to the low purchasing power of the students (better to feed students something even if not healthy (Jensen, 2021) - Children from very impoverished households that have ‘nothing’ are not familiar with basic tools like using tap water which makes implementation of health related education interventions difficult (Obeng, 2016) - When school feeding programs were dependent on parental contributions (monetary, in kind) quality, quantity, frequency of meals served (Roothaert, 2021) - When school feeding programs were dependent on parental contributions, those most in need were not receiving meals, in particular when meal distribution was coupon based (Roothaert, 2021) - When school feeding programs were dependent on parental contributions, but meals were served to all children to avoid stigmatization, parents who contributed were de-motivated (Roothaert, 2021) - When school meals were not for free, project leaders found it difficult to advocate for healthier food, fruits and vegetables which were perceived as more costly and unattainable for the poor communities they were serving (Skar, 2015) - Due to poverty, children do not have much choice in what they eat, as there is no money to make dietary changes (Villiers, 2015) | - School meals help meet the physiological and nutritional needs of children from communities considered as deprived (Cervato Mancuso, 2013) - School meals promote integration between students through their social role (Cervato Mancuso, 2013) - School meals are important in areas with high number of family farmers (as family farmers are the providers) (de Fatima Vilela, 2019) - School meals are important in areas with high level of poverty, in particular in rural areas (de Fatima Vilela, 2019) - School meals are perceived as ‘social welfare interventions’, ex. Although the program is for primary school students, teacher appeals to feed some junior high school students who are in great need (Essuman, 2013) - Children express the perception that if they do not get the school meal, which works best when teachers are involved in the implementation, they may not get food at home that day- due to poverty (Iddrisu, 2017) - School feeding program is perceived as not only benefiting the students within the school, but also the community, as food parcels are sent home to families at end of each week (Mawela, 2018) - School feeding program is satisfying the health needs of children from economically poor families and communities, and as such should be even expanded to include breakfast as well (Mawela, 2018) - Parents of low socio economic background who could not send their children to school due to lack of food are now sending them due to the mid day meal scheme (Mohanty, 2014) - Health in school program is perceived to give poor students access to healthcare and involvement in activities related to food (Moraes, 2018) - Due to economic crisis, children receive less food at home, and thus the school meal becomes their main meal of the day (Studdert, 2004) - Having the school meal for free enables parents to save money during an economic crisis – as they do not give money for snacks, and do not feed the child at lunch time at home (Studdert, 2004) - Providing free school meal has meant children buying less unhealthy snacks from vendors (as they do not receive snack money from their parents (Studdert, 2004) |
| B. Cosmopolitanism | The degree to which an organization is networked with other external organizations. | 18 | - Little cooperation between schools (principals) and cooperatives. Cooperatives were perceived by principals as not reliable, not delivering the food regularly (children whose only meal was the school meal would miss it), and not able to handle the administration which enabled them to be paid (sometimes handled by the principal as there was no other choice) (Beesley, 2013) - Lack of cooperation between schools on developing different educational activities (ex. Activities for lunch ladies performed, no information shared on how this was done with other schools) (Ferreira, 2019) - Lack of communication and cooperation between community members as part of a school management committee in charge of monitoring school feeding, and the cooks, as well as caterers (Iddrisu, 2017) - Lack of trust between teachers and caterers (teachers perceiving the caterers as only interested in profit) (Iddrisu, 2017) - Teachers perceived by students as not involved in the feeding program, not communicating with the caterers (Iddrisu, 2017) - Teachers showing on intrest in the feeding program, no monitoring of delivery and cooking of food (Iddrisu, 2017) - Later delivery of food by suppliers, creates conflict between suppliers, those preparing the food and teacher coordinators (Mafugu, 2021) - System that allows outside vendors to partner with industry that offers financial resources to schools in order to sell unhealthy products in schools (Moise, 2011) - Insufficient participation of health sector in the school context (Moraes, 2018) - No involvement of schools in policy decisions that department of education makes in regard to implementation of the national school nutrition program (Qila, 2014) - Companies (such as Coca Cola, Pepsi, Nestle) provide resources, scholarships, textbooks, computers, infrastructure, training to schools in exchange for branding on school property (Reeve, 2018) - Village and community is in charge of implementing government programs within schools, but they allow vendors to rents school space and often sell unhealthy food (cakes, doughnuts, chips) (Reeve, 2021) - When there was no nutritionist, a committee made of teachers, parents, principals responsible for visiting schools to monitor the feeding program did not implement the visits (Rocha, 2018) - Limited cooperation between teachers and parents influences implementation of school feeding program (Roothaert, 2021) | - Food kiosks (as part of a school based program aimed at preventing obesity) and schools worked under a yearly renewable contract, with a person in charge of coordination of the cooperation (Chavez, 2020) - Head teachers cooperated with caterers to implement a school feeding program (Essuman, 2013) - School feeding program renewed a school-community relationship which was weak (Essuman, 2013) - Mid day meal program improves relationship between school and community, as community participates in the program (Mohanty, 2014) - Cooperation between school, health and education sectors was seen as important in providing for better nutrition guidance as well as dealing with other health needs (Moraes, 2018) - Cooperation of the school and its teachers with the public health representatives, community and parents seen as important throughout the program (Phaitrakoon, 2014) - Schools try to raise funds for the school health and nutrition program from the community (ex. Organizing a cultural program during a festival) (Shrestha, 2019) - Cooperation between schools, local stakeholders, nutritionist ensured that the menus for the school feeding program were developed based on local attainable food (Shrestha, 2020) - School cooperated with kitchen gardeners and farmers to procure local produce (Shrestha, 2020) - To ensure the smooth implementation of the school meals program a committee made of parents, teachers, cooks was set up which cooperated with local cooperatives for procurement of produce (Shrestha, 2020) - School cooperates with local women to prepare snacks, who in return may get some left over food (Studdert, 2004) - Produce for preparation of snacks is purchased from local farmers, some of whom may be the parents of the school children, thus stimulating the local economy (Studdert, 2004) |
| C. Peer Pressure | Mimetic or competitive pressure to implement an intervention; typically because most or other key peer or competing organizations have already implemented or in a bid for a competitive edge. | 7 | - Inside of school, canteens try to sell healthier products, but shops outside of school still sell junk food and attract students (Azizan, 2021) - Kiosks selling food close to schools were not willing to change the products they sold to be more healthy, as they found it may negatively impact their business, as other vendors kept selling unhealthy food (Chavez, 2020) - If the canteen does not sell something that children want (usually unhealthy) they would purchase it outside of school (Girona, 2018) - As long as students could buy unhealthy food outside of school (during school hours, before or after school), principals and food sellers within the school had no motivation to limit the unhealthy food sold within the school (Jensen, 2021) - Teachers reported that as long as vendors outside of schools sold unhealthy food, they could not limit what the canteen was selling (Rachman, 2020) - Difficult to influence what stores around the school were selling, as it may impact their livelihood (Reeve, 2018) - The availability of unhealthy food in the surrounding school environment was influencing canteen commitment for selling healthy food (Reeve, 2021) |  |
| D. External Policy & Incentives | A broad construct that includes external strategies to spread interventions including policy and regulations (governmental or other central entity), external mandates, recommendations and guidelines, pay-for-performance, collaboratives, and public or benchmark reporting. | 14 | - Decisions as to which schools participate in school feeding may be influenced by density of population in the area of the school which is voting for a certain political party (Essuman, 2013) - Farmers had difficulties gaining the needed certificate to sell food to the school feeding program (Ferreira, 2019) - Principals reported lack of communication about the law (healthy snacking initiative) from the side of authorities, some receiving only a notice about the law without any information as to how to implement, while other learned about it in the news (Girona, 2018) - Law did not provide for any legal sanctions for non-compliance with the healthy snacking initiative (Girona, 2018) - Schools found out about the food policy to reduce availability of energy dense foods in the newspaper and from the press, from a letter sent by Ministry of Education, or during regional principal meetings (Jensen, 2021) - Industry lobbying and the education workers union prevent any strong initiative against SSB (Moise, 2011) - Department of Education policy makes it possible for industry to provide infrastructure to schools in exchange for tax incentive (ex. Coca cola foundation) (Reeve, 2018) - Concern among policy makers that any restrictions on certain products (rice, sugar) may hurt the economy of the country (Reeve, 2018) - No plans or resources at national level for implementation of a policy governing food provision in schools (calling for nutrient rich foods) (Reeve, 2018) - No sanctions as part of the policy, in case it is not being followed by school principals (Reeve, 2018) - No sanctions in place for non-compliance with health standards in and around schools (ex. No pressure on food vendors) (Reeve, 2021) - Decision to dedicate more space to nutrition in curriculum should come from senior policy makers and this is not happening as it is in direct competition with other priorities such as sexual health (Reeve, 2021) - Very little guidance on how some policies should be implemented (ex. Just stated that parents should work with school management in regard to school meals) (Roothaert, 2021) - Legislation did not allow for direct relationship with farmers (Scwartzman, 2017) - No program (school health and nutrition program) sustainability if not integrated into the government system (Shrestha, 2019) - Lack of initiative to address the NCD emergency at national level through policy (Skar, 2015) - Gap between international declarations and translation of the same into national, regional, provincial laws and policies (Skar, 2015) - Without funding from government, no incentive to implement (community based school feeding) (Studdert, 2004) | - Due to legislation, food for the school feeding program had to be purchased from family farmers (Ferreira, 2019) - School nutritional programs were introduced at the initiative of the Department of Basic Education to serve learners from poor backgrounds (Mawela, 2018) - Ministry of Health takes the lead in promoting school health (Reeve, 2021) - Decentralization of the meal program made it possible to include local farmers and produce (Schwartzman, 2017) - As food and nutrition security became a priority at national level, various policies were introduced and strengthened in this direction, efforts toward intersectoral coordination were made (Schwartzman, 2017) - As food and nutrition security became a priority, legislative measures were introduced to strengthen not only the ‘biological’, but also ‘social’ aspects of school meals (human right to adequate food, feeding for middle schools, nutrition education in school curriculum, family farming) (Schwartzman, 2017) - Food acquisition program was essential to link school meals with local family farmers (Schwartzman, 2017) - Local procurement only made possible through introducing a law on public calls (Schwartzman, 2017) - Complementarity between the World Food Programme and the school feeding program, without the former, the latter would not be sustainable (Sulemana, 2013) |
| III. INNER SETTING | Includes features of structural, political, and cultural contexts through which the implementation process will proceed. May be composed of tightly or loosely coupled entities (e.g., a loosely affiliated medical center and outlying contracted clinics or tightly integrated service lines within a health system); tangible and intangible manifestation of structural characteristics, networks and communications, culture, climate, and readiness all interrelate and influence implementation. |  |  |  |
| A. Structural Characteristics | The social architecture, age, maturity, and size of an organization. | 16 | - No kitchen on school grounds (Essuman, 2013) - Lack of adequate space to prepare food as part of the healthy snacking initiative (Girona, 2018) - No kitchen in the schools where food could be prepared (Iddrisu, 2017) - No light, sink or running tap water in the kitchen (Mafugu, 2021) - Lack of land and shortage of water make it hard to sustain vegetable gardens (Mawela, 2018) - Lack of proper kitchen, or having a container for a kitchen (Mawela, 2018) - No eating area (Merida Rios, 2019) - No separate room for serving food (Mohanty, 2014) - Tap water not safe or not available at all (Moise, 2011) - No storage and food preparation facilities (Rocha, 2018) - Small kitchen facilities, with no equipment (Rocha, 2018) - Lack of vegetable gardens (Rocha, 2018) - Problems with physical structure of school (Rocha, 2018) - No space for cafeteria (Rocha, 2018) - No storage facilities (Roothaert, 2021) - No kitchen facilities (Roothaert, 2021) - No water (Roothaert, 2021) - No dining space (Roothaert, 2021) - Lack of water (Shrestha, 2019) - No permanent kitchen structure (Sulemana, 2013) - No utensils (Sulemana, 2013) - No water (Sulemana, 2013) - Overall small territory of the school (Villiers, 2015) - Not enough water (Yuvaraj, 2019) | - Private schools had large plots of land, and could contract farmers to plant different vegetables (Roothaert, 2021) - World Food Programme provided tin storage boxes that could be used to store food (Shrestha, 2020) - Vegetable gardens were developed (Skar, 2015) |
| B. Networks & Communications | The nature and quality of webs of social networks and the nature and quality of formal and informal communications within an organization. | 3 | - Communication between school professionals and those working on school feeding not effective (Cervato Mancuso, 2013) | - Cooperation between teachers, parents, students, was key to success of the program (Phaitrakoon, 2014) - At school meetings the principal gives updates on the national school nutrition program to the learners (Qila, 2014) |
| C. Culture | Norms, values, and basic assumptions of a given organization. |  |  |  |
| D. Implementation Climate | The absorptive capacity for change, shared receptivity of involved individuals to an intervention and the extent to which use of that intervention will be rewarded, supported, and expected within their organization. |  |  |  |
| 1. Tension for Change | The degree to which stakeholders perceive the current situation as intolerable or needing change. | 1 |  | - Program was created due to high number of children with cholesterol, diabetes, heart problems, glycaemia, obesity, lack of physical activity (Girona, 2018) |
| 2. Compatibility | The degree of tangible fit between meaning and values attached to the intervention by involved individuals, how those align with individuals’ own norms, values, and perceived risks and needs, and how the intervention fits with existing workflows and systems. Code instances of duplication with existing systems (e.g., between MMT and CPRS, other patient outreach initiatives that target the same patients as AIM) | 11 | - Morning and afternoon school shifts make it difficult to give adequate time for eating (Essuman, 2013) - Nutrition education would be more effective if it were more in line with the regular teaching plan (Gaglianone, 2006) - The school complies with the healthy snacking initiative, to a point- looking for a compromise between the initiative and the demand that sellers are trying to meet (Girona, 2018) - The implementation of a regulatory food policy could be done only to the extent that it did not entirely clash with the profits of the school based kiosk (Jensen, 2021) - A school rule prohibiting the consumption of food during class time hinders the more frequent consumption of fruit and vegetables (Merida Rios 2019) - Lack of time to implement due to a very closed curriculum (Moraes, 2018) - School has too many projects and activities (Moraes, 2018) - Principals found it difficult to have teachers integrate nutrition project in classes through the curriculum (Qian, 2019) - Nutrition education is not part of the curriculum, and thus, there is limited timing to implement it (Rachman, 2020) - Difficult to keep healthy food in canteens when profit of the canteen is threatened (Skar, 2015) | - Canteen operators were familiar with the guidelines of the intervention (healthy school canteen) as they were similar to those from the Ministry (Azizan, 2021) - Teachers stated that their activities are not disturbed by mid day meal scheme (Mohanty, 2014) - When teachers found the nutrition program to be related to health linked subjects as part of the curriculum, they supported making the program permanent (Rachman, 2020) |
| 3. Relative Priority | Individuals’ shared perception of the importance of the implementation within the organization. | 7 | - Canteen operators prioritized profit (Azizan, 2021) - Mental health related activities were prioritized over obesity related activities (Chavez, 2020) - Own schedules of staff was prioritized over program activities (Chavez, 2020) - Difficulty in implementing the nutrition education program due to other priorities (ex. Math, language) (Gaglianone, 2006) - When ‘third parties’ responsible for selling food and beverage, demand and profit were the priority (Girona, 2018) - Coordinator of school nutrition program stated that the syllabus and teaching her learners is the priority (Mawela, 2018) - Health (monitoring how school canteen is managed) is third or last as priority, after curriculum needs, infrastructure (Reeve, 2018) - Nutrition was a low priority for teachers (Reeve, 2021) - Canteen managers had a lot of agency in how they ran canteens, and did not prioritize nutrition, but rather perceived themselves as business people (Reeve, 2021) | - When healthy eating was prioritized, it was complemented by growing trees, nutrition promotion materials (Reeve, 2021) |
| 4. Organizational Incentives & Rewards |  | 3 | - Sales dropped due to implementing a regulatory food policy, and the school had to lower the rent for the sellers on school property (Jensen, 2021) - Low commitment of coordinators of a school nutrition program, as it is seen as extra work without pay (Mawela, 2018) - Salaries paid to cooks are not enough (Mohanty, 2014) |  |
| 5. Goals and Feedback |  | 2 | - Even though the healthy snacking initiative and its goals were shared by principals with canteen, they were not followed (Girona, 2018) - Kiosks sellers on school grounds claimed not to have been informed of what to sell and what not to sell (Jensen, 2021) |  |
| 6. Learning Climate |  |  |  |  |
| E. Readiness for Implementation | Tangible and immediate indicators of organizational commitment to its decision to implement an intervention. |  |  |  |
| 1. Leadership Engagement |  | 11 | - School that did not fully implement an obesity prevention program had a school director who gave low priority to program activities (Chavez, 2020) - When principals did not have interest in the topic of nutrition, there was no additional time and effort invested in implementing the healthy snacking initiative (Girona, 2018) - Not all principals supportive of nutrition education program (Qian, 2019) - When principals were not involved, the sustainability of the program was in question (Rachman, 2020) - Principal had little power when the canteen vendor was family of school committee members (Reeve, 2021) | - School directors and teachers were key to implementing a school based program aimed at preventing obesity (Chavez, 2020) - Compliance with healthy snacking initiative depended on how much principals were interested in the topic of nutrition (Girona, 2018) - Principals dedicated to nutrition and the healthy snacking initiative in some schools were already taking action before the initiative, and took additional measures during (banning unhealthy food, taking it away from students, giving it back at the end of day) (Girona, 2018) - Principals had an important role, they were aware of, in management of school nutrition programs (not day to day, but for ex. Hiring food handlers, assigning a school nutrition coordinator) (Mawela, 2018) - School principals key in implementing the program and for its sustainability (Phaitrakoon, 2014) - When principals played an important role in nutrition education program, teachers were motivated to adopt nutrition and health related programs (Rachman, 2020) - Role of the principal key to sustainability of school food policies, and introducing new nutrition related programs such as school canteen, nutrition education vegetable program for undernourished children (Reeve, 2018) - Principal key to success of enforcing healthy eating standards in schools (Reeve, 2021) - Principal key to success of school based health promotion projects (Skar, 2015) - Principal is key in motivating all other school employees (Villiers, 2015) - School administration not allowing unhealthy food on school property is of help (Yuvaraj, 2019) |
| 2. Available Resources |  | 24 | - Insufficient number of workers in canteen (Azizan, 2021) - Too much time spent on eating and cleaning up by staff and pupils (Essuman, 2013) - Capacity building (nutrition knowledge, use of technology) for using the school meals planner tool needed (Fernandes, 2016) - No financial resources for educational activities (Ferreira, 2019) - Lack of time of teachers to attend meetings after school hours (Gaglianone, 2016) - Rent paid by kiosks on school property is used to supplement school funding for different activities (like school meals, infrastructure) (Jensen, 2021) - Low attendance of training for food handlers and service providers due to logistical issues (transport not organized, venue not accessible) (Mafugu, 2021) - Not fulfilling certain roles (not anticipated by project but recognized by stakeholders), due to time limitations (ex. Principals following up on what to do when there are food allergies) (Mawela, 2018) - No time to do both nutrition coordination and teaching (Mawela, 2018) - Need on training as to how to manage the school nutrition program by teachers (ex. How to avoid teachers leaving class in order to receive food, or check on food preparation) (Mawela, 2018) - Doing additional activities, such as having and maintaining a vegetable garden adds to the managerial load for principals, for which they do not have time (Mawela, 2018) - Lack of time for eating fruit and vegetables reported by teachers (Merida Rios, 2019) - Arrangements linked to the mid day meal falls on teachers (Mohanty, 2014) - General reference to lack of resources (Moise, 2011) - Lack of external professionals to assist with health in school program (Moraes, 2018) - No training for school employees (Moraes, 2018) - Not enough teachers considering the large number of students, for implementation of health education activities (Obeng, 2016) - Not enough persons with appropriate background (ex. In under nutrition) (Obeng, 2016) - Lack of teaching materials (Obeng, 2016) - Teachers not having time for paperwork linked to program on top of all other responsibilities (Phaitrakoon, 2014) - Additional time demands on principals and teachers (Qian, 2019) - Not enough training especially for teachers with backgrounds not in nutrition (Rachman, 2020) - Time constrains, considering nutrition is not part of the curriculum (Rachman, 2020) - Financial resources of the school (teacher salaries, for the school feeding program) are directly dependent on profitability of canteens, thus restricting the possibility to enact rules about sale of processed foods (Reeve, 2018) - Some resources of the schools come from sponsorships (adopt a school program) by food companies (Coca cola) (Reeve, 2018) - Maintenance of infrastructure done with revenue from canteens, making it hard to put restrictions on unhealthy food they sell (Reeve, 2021) - Schools lack equipment such as scales, thermometers, blenders (Rocha, 2018) - Lack of training for cooks (Rocha, 2018) - Shortage of educational materials to work on eating habits (Rocha, 2018) - Great variability of financial and in-kind contribution schools receive from external sources for the implementation of the home grown school feeding program (reasons for this unknown) (Roothaert, 2021) - Training lacking or insufficient frequency for teachers implementing the school health and nutrition program (Shrestha, 2019) - Schools reporting lack of resources to sustain the program (Shrestha, 2019) - Lack of time (Villiers, 2015) - Need of an external person to give guidance on some aspects of program (such as gardening) (Villiers, 2015) - Lack of training for teachers on some aspects of health promoting schools framework (Yuvaraj, 2019) | - Workshops with lunch ladies (Ferreira, 2019) - When training was given to teachers, especially those with no background knowledge in nutrition, it was found very beneficial for implementation of nutrition education program (Rachman, 2020) - Cooks hired for the implementation of the home grown school feeding program were also trained in ingredient use, estimation of quantities, food preparation, hygiene practices and food storage (Shrestha, 2020) - Parents received training on nutrition, food preparation and gardening (Shrestha, 2020) - Teachers received training in how to integrate program materials into the curriculum (Shrestha, 2020) - Teachers offered materials on integration of HealthKick intervention in curriculum (Villiers, 2015) |
| 3. Access to Knowledge and Information |  | 9 | - Materials to teach nutrition education needed (Gaglianone, 2006) - Principals had difficulty identifying recommended products by Ministry of Health due to lack of clear guidelines (Girona, 2018) - Lack of information by students, parents, and supervisors, lack of involvement and information by teachers (Iddrisu, 2017) - No information on what sellers on school property are allowed to sell (Jensen, 2021) - Learning materials that would help the understanding of students missing (Rachman, 2020) - No clear guidance on what the policy means, principals, teachers had to interpret on their own (Reeve, 2018) - No clarification by officials in regard to the home grown school feeding program, free education (Roothaert, 2020) | - External public health staff invited in school to explain program (Phaitrakoon, 2014) - Teachers had a guidebook that they could use to implement home grown school feeding (approved by curriculum development center) (Shrestha, 2020) |
| IV. INDIVIDUAL CHARACTERISTICS | Individuals involved with the intervention and/or implementation process. Code specific roles related to champion, opinion leaders, formally appointed implementation leaders, and external change agents under the appropriate code under PROCESS. Mostly, these codes involve people who are the target of the intervention (primarily users). |  |  |  |
| A. Knowledge & Beliefs | Individuals’ attitudes toward and value placed on the intervention as well as familiarity with facts, truths, and principles related to the intervention. | 20 | - Teachers believe that students do not have understanding about healthy eating (Azizan, 2021) - Teachers believe students do not like vegetables, healthy beverages and prefer sweet beverages and unhealthy food (Azizan, 2021) - Perception of principals and pedagogical coordinators that children do not eat some foods because they are not familiar with them (Cervato Mancuso, 2013) - Principals lacked knowledge about the healthy snacking initiative (Girona, 2018) - Principals perceived compliance was higher than actual compliance of the school with the healthy snacking initiative (Girona, 2018) - Principals belief that students do not like healthy food and prefer unhealthy food (cookies, snacks, soda) (Girona, 2018) - Principals belief that family habits influence what students eat (Girona, 2018) - Principals and in –school kiosk workers belief that it is the family that is responsible for establishing healthy eating habits of students, and that the school can only have a supporting role (Jensen, 2021) - Both principals and in-school kiosks workers had lack of knowledge about the guidelines as part of the food policy to limit energy dense foods in high schools (Jensen, 2021) - Both principals and in-school kiosks workers had misconceptions about guidelines (ex. Baked potato chips and small chocolate candies are allowed) (Jensen, 2021) - Both principals and in-school kiosks workers expressed belief that adolescents do not like healthy products which makes it hard to adhere to the food policy (Jensen, 2021) - Different stakeholders belief that home environment, and home made unhealthy products (such as sugary beverages) are difficult to regulate (Moise, 2011) - Teachers belief that parents do not provide nutrition diets at home, and lack knowledge on nutrition (Rachman, 2020) - Lack of knowledge on nutrition among principals (Reeve, 2018) - School based food vendors lack of understanding of nutrition as well as guidelines (Reeve, 2021) - Belief by policy makers and implementers that children prefer unhealthy foods (Reeve, 2021) | - Principals and pedagogical coordinators believe although it is harder to change parental eating habits, it is possible to change child eating habits (Cervato Mancuso, 2013) - Belief by principals and pedagogical coordinators that it is the responsibility of the school to feed the children with high quality products (Cervato Mancuso, 2013) - Belief by principals and pedagogical coordinators that feeding children with food that is rich in vitamins, minerals, promotes health and welfare (Cervato Mancuso, 2013) - Belief by various stakeholders linked to national school meals that the program brings dignity to the community, and increase income for farmers (de Fatima Vilela, 2019) - Belief by teachers and head teachers that the school feeding program increases attendance of children (Essuman, 2013) - School nutrition program coordinators belief that grades, sport performance and attendance has improved due to the program (Mawela, 2018) - Coordinator belief that breakfast in class as part of the program has helped punctuality and discipline of learners (Mawela, 2018) - Head master, teacher and parents belief that school attendance has increased thanks to mid day meal scheme (Mohanty, 2014) - Principals and teachers belief that health in school program is positively changing eating habits at home (Moraes, 2018) - Principals and teachers belief that educating about healthy eating is important as children carry it to adulthood (Moraes, 2018) - Teacher stating that due to the project, had an opportunity to lunch with students, and have more vegetables (Phaitrakoon, 2014) - Teachers stated their motivation increased when there was positive feedback from students (Rachman, 2020) - Teachers belief that school feeding is a basic human right (Roothaert, 2021) - Perception by stakeholders that school health and nutrition program has a positive impact on students, schools and communities (Shrestha, 2019) - Principals and teachers aware of objectives of the program (Shrestha, 2020) - Belief among teachers that the program makes it easier for children to learn the topic (Shrestha, 2020) - Principals, teachers and parents belief that the program increased attendance, improved dietary and hygiene practices, and grades (Shrestha, 2020) - Head teachers belief that the school feeding program improved punctuality and attendance (Sulemana, 2013) - HealthKick intervention made teachers try to improve their own healthy (Villiers, 2015) - Teachers and principals perception that parents prepare healthy food for their children reinforcing the nutrition education sessions (Yuvaraj, 2019) |
| B. Self-efficacy | Individual belief in their own capabilities to execute courses of action to achieve implementation goals. | 2 | - Teachers not understanding the requirements of the program (Phaitrakoon, 2014) - Teachers expressed difficulties in delivering parts of the nutrition education program (ex. Concepts) (Rachman, 2020) |  |
| C. Individual Stage of Change | Characterization of the phase an individual is in, as he or she progresses toward skilled, enthusiastic, and sustained use of the intervention. | 1 | - Teachers feeling they cannot implement, due to lack of training (Yuvaraj, 2019) |  |
| D. Individual Identification with Organization | A broad construct related to how individuals perceive the organization and their relationship and degree of commitment with that organization. INCLUDE Organizational citizenship: how organizational identity is taken on and whether indivs are willing to put in extra effort, talk well of the organization, and take risks in their organization. Organizational justice: perception of distributive and procedural fairness in the organization. Emotional exhaustion: burnout. Etc. |  |  |  |
| E. Other Personal Attributes | A broad construct to include other personal traits such as tolerance of ambiguity, intellectual ability, motivation, values, competence, capacity, and learning style. | 4 | - Teachers lack of motivation due to other priorities (Gaglianone, 2006) - Teachers lack of professional qualifications to teach the topic (Obeng, 2016) - Teachers lack of nutritional or health related background needed to teach nutrition education (Rachman, 2020) - Lack of teachers with knowledge of health issues (Shrestha, 2019) |  |
| V. PROCESS | Activities aimed to achieve individual and organizational level use of the intervention as designed. Process may be an interrelated series of sub-processes that do not necessarily occur sequentially. These sub-processes may be formally planned or spontaneous; conscious or subconscious; linear or nonlinear. |  |  |  |
| A. Planning | The degree to which a scheme or method of behavior and tasks for implementing an intervention are developed in advance and the quality of those schemes or methods. | 4 | - Guidelines related to regulatory food policy to reduce availability of energy – dense foods just provided, without any planning or guidance (Jensen, 2021) - Need for a yearly plan to incorporate the health in school program in yearly activities of the school (Moraes, 2018) - General comment on lack of planning as a barrier (Villiers, 2015) | - Year action plans where the educational component as part of a program aimed at obesity prevention was described in detail was available, together with tools to be used (Chavez, 2020) |
| B. Engaging | Attracting and involving appropriate individuals in the implementation and use of the intervention through a combined strategy of social marketing, education, role modeling, training, and other similar activities. |  |  |  |
| 1. Opinion Leaders | Individuals in an organization who have formal or informal influence on the attitudes and beliefs of their colleagues with respect to implementing the intervention | 2 | - Community opinion leaders did not engage in a school feeding program, due to lack of involvement of the teachers (Iddrisu, 2017) | - Civil society and professionals from health, nutrition, food security discussed family farming as part of the school feeding program when gathering at various events (such as forums, conferences) (Schwartzman, 2017) |
| 2. Formally Appointed Implementation Leader | Individuals from within the organization who have been formally appointed with responsibility for implementing an intervention as coordinator, | 4 | - Caterers were appointed as having a lead role in the management of a school feeding program, however, as external to the school, and without cooperation from the teachers they faced significant challenges in implementation (Iddrisu, 2017) - Lack of teacher on health education (Yuvaraj, 2019) | - Nutrition coordinator as part of the school nutrition program has the responsibility of placing orders, checking food quality, and making sure those in need get the food (Mawela, 2018) - One teacher in charge of monitoring implementation, with assistance/support of other teachers (Phaitrakoon, 2014) |
| 3. Champion | “Individuals who dedicate themselves to supporting, marketing, and ‘driving through’ an [implementation],” overcoming indifference or resistance that the intervention may provoke in an organization. |  |  |  |
| 4. External Change Agent |  | 19 | - Shop operators cannot sell fresh foods (Azizan, 2021) - The government loans given to cooperatives which were supplying food to schools were given to all who were part of such cooperative, regardless of the quality of their service, or whether or not they gave back the loan (which was seen as a grant, with no obligation to repay) (Beesley, 2013) - Receiving funds regardless of work done by cooperatives created conflict, tension, mistrust and suspicion among members (Beesley, 2013) - Training was offered to cooperative members only for a limited time period, with no future support structure set up and had significant challenges to implement as some did not know how to read or write (Beesley, 2013) - Cooperative members did not submit required documentation as part of receiving the government loan (Beesley, 2013) - Family farmers tend not to attend or participate in public calls (de Fatima Vilela, 2019) - No training offered to family farmers (de Fatima Vilela, 2019) - Community members who are part of a school committee implementing a school feeding program on a voluntary basis do not engage, and do not attend meetings as the cost of doing so versus doing their own economic activity has increased (Essuman, 2013) - Not enough nutritionist to participate in the national school feeding program, whose role is monitoring, planning, execution – generally supporting the schools in implementing the program (Ferreira, 2019) - External members of School Feeding Board (responsible for school visits to monitor implementation and speak to the children) facing challenges to participate due to lack of training and transportation (Ferreira, 2019) - Overload of the nutritionist responsible for assisting implementation of the school feeding program with administrative and bureaucratic tasks (Ferreira, 2019) - Supervisors who have the role of supervising the implementation of the curriculum as part of the Ministry of Education, were also responsible for monitoring the school feeding but were not aware of the program or their tasks (Iddrisu, 2017) - Strong lobbying by industry to limit any action in regard to sugar sweetened beverages in schools (Moise, 2011) - Lack of communication between health and education in implementing the health in school program (intersectorality) (Moraes, 2018) - Insufficient technical support for family farmers, especially by municipal agriculture departments (Mossmann, 2017) - Food companies working together in lobbying to ensure no limitation to selling unhealthy food in schools are introduced (ex. Pepsi and Coca cola working together in this regard) (Reeve, 2018) - Ministry of Health passing on the responsibility of implementing standards in regard to nutrition in schools to Ministry of Education, and vice versa (Reeve, 2021) - Lack of nutritionist responsible for monitoring and assisting implementation of the school feeding program (Rocha, 2018) - Family farmers lacking transportation to make deliveries to schools (Rocha, 2018) - Community development officers responsible for participatory community based projects did not take part in raising money for school feeding (Roothaert, 2021) - Limited coordination between Ministry of Health, Ministry of Education, which led to lack of planning, sustainability and scale up of school health and nutrition program (Shrestha, 2019) - Limited participation of Ministry of Education in the school health and nutrition program (Shrestha, 2019) - No training for the cooks- volunteers from the women’s association (Studdert, 2004) - District implementation committees not active in all schools (Sulemana, 2013) - Schools did not purchase from local farmers, and thus did not boost their production (Sulemana, 2013) - Community members, chiefs, opinion leaders had no information on the school feeding program (Sulemana, 2013) | - Where nutritionists external to the school were involved in the health in school program, they had an important role in making menus, working with families on diet, education in regard to healthy eating (Moraes, 2018) - Intersectorality and development of cooperation, dialogue, between family farmers and other actors such as Secretariat for Agriculture and Fisheries, as key to dealing with implementation challenges (Mossmann, 2017) - Provision of some training and education to family farmers in regard to technical issues (seeding, harvest, production, transportation) (Mossmann, 2017) - Organisation of farmers into cooperatives as key to handle the demands of the program (Mossmann, 2017) - An advocacy group as part of parliament focusing on healthy lifestyles, was key in taking actions against schools not meeting sanitation standards (Reeve, 2021) - Officials from education sector gave importance to nutrition, and participated in some events and actions in this direction (Reeve, 2021) - Social movements and family farmers lobbied for participation of family farmers in the school feeding program (Schwartzman, 2017) - Due to involvement of local government administrators, family farmers were involved in school feeding even before this was enabled by the law (Schwartzman, 2017) - Food and nutrition security national council (CONSEA) had a crucial role on linking family farming with school feeding, and on many positive developments having to do with school feeding (Schwartzman, 2017) - Training offered cooperatives on accounting, billing monitoring, useful in teaching them these needed skills to participate in the home grown school feeding program (Shrestha, 2020) - Provincial and regional directorates as part of the Ministry of Education more active in the school based health promotion projects (in comparison to the central level (Skar, 2015) - Volunteering members of the village women’s association had crucial role in preparing the meals as part of community based school feeding, motivated by social aspects, learning new cooking methods and recipes, personal satisfaction with the task of cooking for children (Studdert, 2004) - School feeding program benefited local farmers (Studdert, 2004) - Other community members (such a village leaders, midwives) monitored the implementation of the school feeding program, whether or not the children ate, the hygienic practice of cooks, and gave advice based on that (Studdert, 2004) |
| 5. Key Stakeholders (Staff) |  | 10 | - Teachers emphasized long working hours as a barrier (Chavez, 2020) - Although yearly implementation plans did exist for the school based program aimed at preventing obesity, there were no descriptions of roles of different implementers, which was a barrier (Chavez, 2020) - Teachers are not involved in any decision making that has to do with the national school nutrition program, they are only informed of decisions by the principal, who is informed by the Department of Education (Qila, 2014) - Resistance of cooks to some activities as part of the national school feeding program (Rocha, 2018) - Teachers not involved in the home grown school feeding program due to mistrust from the side of parents and not wanting to be involved (Roothaert, 2021) | - Schools had health and nutrition committees responsible for monitoring the cafeteria (but not kiosks) (Jensen, 2021) - School management committees and food committees are formed to assist the mid day meal scheme (ex. Prepare food list, choose vegetables, check cleanness) (Mohanty, 2014) - The school management committee cooperates with headmasters of the school in implementation of the mid day meal scheme (Mohanty, 2014) - The school management committee tries to convince students (and parents) who have dropped out or are often absent to come back to school (Mohanty, 2014) - Efforts were made to have teachers participate in the nutrition education program (no further description given) (Qian, 2019) - Involvement of school management, students, teachers, parents, communities as stakeholders in school interventions is important (Skar, 2015) - School principal, teachers, women’s associations, village leaders and midwives were identified and participated in the implementation of the community based school feeding (Studdert, 2004) - Teachers conduct sessions on healthy dietary practices during class hours (Yuvaraj, 2019) |
| 6. Innovation Participants (Patients) |  | 23 | - Adolescents (boys and girls) as part of the healthy school canteen expressed views that there is lack of variety of healthy food, fruit and vegetables, unhealthy food is more attractive, and canteen food is often oily, too salty, too spicy, with low hygiene conditions (Azizan, 2021) - Parents were informed of activities as part of the school based program aimed at preventing obesity through notes on school diary of their children, however, parental attendance at events was low due to work obligations (Chavez, 2020) - Students could not attend due to the sessions being out of school hours (Chavez, 2020) - Insufficient involvement of parents in nutrition education activities (Gaglianone, 2006) - Challenge in involving parents due to their daily work schedules (Moraes, 2018) - Generally low participation in health related activities of some parents, and transference of some health related responsibilities in regard to the child to the school (ex. Sending the child to school even when sick, knowing that the school will take care of them) (Moraes, 2018) - Lack of engagement of parents as part of the health education activities in schools, and in some cases not providing information in regard to their children (Obeng, 2016) - Lack of engagement of parents in nutrition education program due to being unfamiliar with such programs (Qian, 2019) - Parents not invited and involved in decision making having to do with the national school nutrition program (Qila, 2014) - Food as part of the national school feeding program was not eaten by more than half of students, and their parents stated they sent food for home as they thought the school food was bad (Rocha, 2018) - Parents not aware that school meals are not covered by the free education policy and are thus dependent on their contributions (Roothaert, 2021) - Parents not seeing vegetables as something nutritious but something that you eat out of poverty (Roothaert, 2021) - No participation of parents in planning activities as part of HealthKick intervention (Villiers, 2015) | - Teacher encouragement, guidance, motivation of students during meals was essential for the implementation of the school feeding program, and when needed, and teachers saw waste of food, they spoke to the students about it (Cervato Mancuso, 2013) - Students looked forward to coming to school because of the school meal, especially on days with their favourite meal, some stating that it increased activity and attentiveness in class (Essuman, 2013) - Families were informed of the school meals planner package via radio as well as vans broadcasting jingles while driving through communities (Fernandes, 2016) - Group activities and lectures for parents and students were implemented by nutritionists, teachers, educators (Ferreira, 2019) - Educational activities targeted at children (food related topics integrated in school subjects, cooking workshops, vegetable garden) and parents (discussing healthy eating, snacking, sharing notebook with recipes) (Girona, 2018) - Children arrived early to school due to the breakfast in class (Mawela, 2018) - All children (regardless of need) ate meals due liking the food (Mawela, 2018) - The mid day meal scheme improved relationship between school and parents, allowing for parents to participate in meal preparation (Mohanty, 2014) - Children found the food from the program acceptable, liked sharing it (Mohanty, 2014) - Parents of poor background had positive view of the scheme (Mohanty, 2014) - The scheme was perceived by parents to improve some habits such as washing hands, respect for elders (Mohanty, 2014) - Nutritionist speaking with the parents was more helpful than the teachers talks, due to credibility on the topic (Moraes, 2018) - Some parents participate in health in school program activities, as they want to be involved in the development of their children (Moraes, 2018) - Lectures as part of the health in school program available for parents as well (Moraes, 2018) - Parental involvement seen as important by those external to the program but involved in its implementation within the school (Moraes, 2018) - Invitation of health education experts at every parent meeting, and sending of newsletters to parents on issues of obesity and weight control (Phaitrakoon, 2014) - Students received information on weight control as part of different subjects (Phaitrakoon, 2014) - Students liked the healthy alternatives and meals offered by cooks at their school (Phaitrakoon, 2014) - Students perceived healthy foods to contribute to good health, mood, weight control, concentration, and being cheerful (Phaitrakoon, 2014) - Parent – child joint activities to engage parents as part of nutrition education program (Qian, 2019) - Attempts to reach parents through social media (Qian, 2019) - Students had a positive view of the nutrition education program (Rachman, 2020) - Those students who did eat the school food (less than half) considered it good/very good (Rocha, 2018) - Awareness raising activities by school food committees trying to explain to parents that the meal is not covered by the new free education policy (Roothaert, 2021) - Organization of food and nutrition fairs by teachers and principals, for parents and children, all involved expressed positive views of the events (Shrestha, 2020) - Students participated in development of materials such as pamphlets, to share the message of school based health promotion project (with references to locally available foods) (Skar, 2015) - Students used as change agents as part of the program (ex. Measuring BMI) (Skar, 2015) - Parents were invited to cooking competitions (Skar, 2015) - Members of parents association coming to schools several times per year to taste the food as part of school feeding (Studdert, 2004) - Students involved in promoting hygiene and community collaborative measures (Yuvaraj, 2019) - Parents followed healthy habits at home (Yuvaraj, 2019) |
| C. Executing | Carrying out or accomplishing the implementation according to plan. | 12 | - Delayed government payments to producers, cooperatives, which due to lack of capital by the latter leads to not delivering or cancelling orders (de Fatima Vilela, 2019) - Low participation of family farmers in the school meal program as they cannot deliver the goods (de Fatima Vilela, 2019) - Caterers as part of the school feeding program are expected to pre-finance the meals, and get reimbursed by government, however, government reimbursements are delayed and caterers have to resort to taking loans (Essuman, 2013) - Lack of enforcement of food policy by principals, so that on site sellers do not loose profit (Jensen, 2021) - Principals submit documentation linked to school nutrition program late, which causes additional delays in payments from the government, and further results in meals not being delivered to learners (Mafugu, 2021) - Late deliveries of gas for cooking resulted in meals not being prepared (Mafugu, 2021) - Irregular payments linked to mid day meal scheme (Mohanty, 2014) - Delayed payments by government to family farmers (Mossmann, 2017) - School food policies exist, but are not being implemented (Reeve, 2018) - Most schools did not receive a visit by nutritionist as part of national school feeding program (Rocha, 2018) - Most cooks did not receive training as part of national school feeding program (Rocha, 2018) - Meals made by mixing all ingredients at the discretion of the cooks, without any use of guidelines, manuals or menus (Rocha, 2018) - Insufficient quantities of fruit and other produce delivered (Rocha, 2018) - Logistical difficulties with delivery of produce (Rocha, 2018) - Insufficient supply of macro and mic nutrients in school meals compare to what is recommended (Rocha, 2018) - Some occurrence of funds embezzlement by members of food committee as part of home grown school feeding program (Roothaert, 2021) - Food committee members who are suppose to supervise implementation (such as storage, preparation, provision of food) show up late or not at all (Roothaert, 2021) - Delay in reimbursements from government as part of community based school feeding (although this did not disturb implementation due to efforts by implementers, such as women’s association member who in those cases borrow money from school cash) (Studdert, 2004) - Long delays in payment of funds as part of school feeding program (Sulemana, 2013) - Schools did not follow procurement procedures due to delayed release of funds (ex. They had only one trusted supplier, who would be willing to supply on credit) (Sulemana, 2013) | - Schools reported implementing changes to sales of sugar sweetened beverage and other high energy food, or decreasing sugar in drinks prepared on site, due to new government food policy (Jensen, 2021) - Hygiene practices implemented well as part of home grown school feeding program (Shrestha, 2020) |
| E. Reflecting & Evaluating | Quantitative and qualitative feedback about the progress and quality of implementation accompanied with regular personal and team debriefing about progress and experience. | 7 | - No clear information on who should monitor the implementation of guidelines as part of food policy, with only sporadic monitoring by principal (Jensen, 2021) - Lack of monitoring from government agencies as key factor why school food policies and marketing restrictions were not being implemented (Reeve, 2018) - Lack of resources and lack of criteria for monitoring from the government agencies (Reeve, 2018) - Some complementary activities to the food policy (ex. Purchase of local foods) were not monitored (Reeve, 2021) - No formal evaluation of implementation of standards in schools conducted (Reeve, 2021) | - Control from authorities if the healthy snack initiative is being implemented a key factor for compliance of schools (Girona, 2018) - Some control by Ministry of Health in regard to implementation of guidelines as part of food policy (Jensen, 2021) - School management committee involved in monitoring mid day school meal implementation (Mohanty, 2014) - When Department of Agriculture made effort to meet with family farmers, and discuss how things went with the school meal program, what the challenges were and what can be done about it, this enabled positive changes and improvement (Mossmann, 2017) - Government organized a clear monitoring process (with visits by nutritionists to schools) to ensure implementation of the standards for school food, with reports being fed back to principals who found it a useful reminder of the policy, as well as to health and education government bodies (Reeve, 2021) - Award system for schools who receive positive reports by monitoring visits, and reprimands for those who do not (Reeve, 2021) - Principals took initiative and conducted monitoring in addition to the government, going as far as cancelling contracts of canteen operators who did not comply with food policy (Reeve, 2021) - Technical supervision of implementation of national school feeding program by nutritionists implemented well as long as there were enough nutritionists employed (Rocha, 2018) |
