# Supplementary material for: Barriers and facilitators to implementation of nutrition-related actions in school settings in low- and middle-income countries (LMICs): a qualitative systematic review using the Consolidated Framework for Implementation Research (CFIR)
Source: Implement Sci Commun. 2023 Jun 27;4:73. doi: 10.1186/s43058-023-00454-y (PMC10294384; doi:10.1186/s43058-023-00454-y)
Supplement: Supplementary file 4 — Additional file 4. ENTREQ Checklist. [file 43058_2023_454_MOESM4_ESM.docx]

ENTREQ Checklist [1]

| **No** | **Item** | **Guide and description** |
| --- | --- | --- |
| **1** | Aim | The aim of this paper is to systematically review research on barriers and facilitators that influence implementation of nutrition related interventions/policies/programs in the school setting in LMICs. The focus will be specifically on qualitative research, as most appropriate for exploring and understating barriers and facilitators in depth. |
| **2** | Synthesis methodology | For extraction of qualitative data relating to barriers and facilitators to implementation, we followed the principles of qualitative meta synthesis and thematic synthesis of qualitative research in systematic reviews [2, 3]. All text from the primary articles which was referring to any identified barriers and facilitators was copy-pasted in its original form in a separate word document for each article included in this review. Word documents were then uploaded to NVivo for deductive coding based on the CFIR, using the codebook provided on the relevant CFIR website [4, 5]. Coding was done by one reviewer with previous experience of using the CFIR in the context of qualitative systematic reviews (B.M.). Once coding was completed the text under each construct for all papers included in the review was extracted into a matrix following the principles of framework analysis [6]. Subsequently the text under each construct was summarized and categorized into barriers and facilitators. |
| **3** | Approach to searching | The search was a pre-planned comprehensive search of all available studies. |
| **4** | Inclusion criteria | Implementation of nutrition-related activities in school settings, pre-experimental designs and qualitative studies, from earliest available to October 2021 are included. Full text, peer reviewed and published articles/book chapters are included. There are no language limitations. Geographical focus on LMICs. |
| **5** | Data sources | The following databases covering health, psychology, education and interdisciplinary subjects were searched: EMBASE, ERIC, Medline, Global Health and PsycInfo (all on Ovid), Scopus (Elsevier), the Web of Science Social Sciences Citation Index and Global Index Medicus from World Health OrganizationTo identify studies in LMICs, the LMIC filter from Cochrane was used, except in Global Index Medicus where a simpler search strategy was chosen due to search functionality. We searched for both database specific subject headings and in the fields for title, abstract and author keywords. |
| **6** | Electronic Search strategy | The search consisted of several synonyms for interventions on nutrition, in combination with synonyms for school related topics and implementation. |
| **7** | Study screening methods | The screening process was conducted in two stages, based on the inclusion and exclusion criteria. The first stage was a title and abstract screen, with all titles and abstracts screened by two reviewers (B.M. screened all, P.A., M.G., P.O.I. were second reviewers, while N.L. resolved all conflicts). In case of doubt about a title or an abstract, or when the abstract was missing, a study was included for a full text screen. The second stage was a full text screen with all texts screened by two reviewers (as for abstract screening). At this stage, articles which were systematic reviews, dissertations, protocols, conference abstracts, not in English, or the full text was not available were excluded. |
| **8** | Study characteristics | The most common type of nutrition-related action described in the included articles was the provision of a school meal (44%), followed by nutrition-related policies coming from the international or national level (28%) and nutrition-related education programs (19%). Studies were most commonly conducted in Brazil (25%), South Africa (17%) and Ghana (14%). It should be noted that in 14 out of the 36 papers included in this review, it was clearly stated that the studied action was targeting children in areas of low socio-economic background. The most common data collection method across papers was interviews (81%), however, often in combination with other methods such as observation (31%), focus group discussions (25%), questionnaire interviews with qualitative data (19%) and document analysis (14%). Finally, the participants of the primary articles were most often school staff (teachers, principals, canteen staff) (78%), followed by persons external to the school setting (such as nutritionists and suppliers) (53%), children (33%) and parents (22%). |
| **9** | Study selection results | A total of 6775 records were identified based on the search, with 4253 remaining for title and abstract screen after deduplication. Of the 4263 records, 4030 were excluded based on title and abstract screen. Of the 223 remaining for full text screen, 187 were excluded with reasons. The most common reason was that there were no barriers and facilitators identified. A final 36 articles were included in this review. |
| **10** | Rationale for appraisal | We conducted an appraisal of the methods of each article included. |
| **11** | Appraisal items | For the purpose of this review, two checklists specifically designed for quality appraisal of qualitative primary studies were combined and used. The Critical Appraisal Skills Programme (CASP) list which consists of ten questions was used as a basis for the evaluation [7]. However, CASP was supplemented with two additional questions on use of a theoretical framework, and the use of relevant references linked to the topic, from the ‘Guidelines for authors and reviewers of qualitative studies’ [8]. Thus, all included papers were evaluated on a total of 12 points. |
| **12** | Appraisal process | Five of the total included articles were evaluated by two reviewers (B.M. and M.W.) while all remaining were evaluated by one reviewer (B.M.). |
| **13** | Appraisal results | The articles included were of mixed quality. All of the included articles had a clear statement of aims and a qualitative method was considered to be the appropriate choice according to that aim. However, not all discussed why a qualitative method was chosen. Several articles were found to miss key information in regard to the participants such as sample size and recruitment. Very few articles discussed the relationship between researcher(s) and participants, and in eight articles ethical issues were not mentioned at all. Six articles did not discuss the process of analysis. Finally, 22 articles lacked any reference to a theoretical framework. However, as all articles were evaluated to have a clear statement of findings, and those findings were evaluated to be of value, no articles were excluded based on the quality appraisal. |
| **14** | Data extraction | Data related to the characteristics of the articles and qualitative data related to barriers and facilitators was extracted. Data extraction for ten articles was completed by two reviewers (B.M. and P.A., P.O.I., M.W., N.L.) while data for the remaining articles was extracted by one reviewer (B.M.). In regard to characteristics of the articles, the following information was extracted in an excel sheet: first author and year of publication, title, aim, action description, geographical focus, data collection methods, sample size and participants and school type. For extraction of qualitative data relating to barriers and facilitators to implementation, we followed the principles of qualitative meta synthesis and thematic synthesis of qualitative research in systematic reviews [2, 3]. |
| **15** | Software | Rayyan (for title and abstract screen); COVIDENCE (for full text screen); NVivo (for deductive analysis) |
| **16** | Number of reviewers | All co-authors participated in screening and data extraction. B.M. and M.W. conducted the quality appraisal. |
| **17** | Coding | Coding was done deductively in NVivo, using the CFIR. Coding was done by one reviewer with previous experience of using the CFIR in the context of qualitative systematic reviews (B.M.). (please also refer to point 2). |
| **18** | Study comparison | Coding and analysis were conducted using the pre-developed coding structures based on CFIR. |
| **19** | Derivation of themes | Deductive coding was conducted. |
| **20** | Quotations | Example quotation from the sub-construct available resources, reflecting the views of the author:  ‘Given that some schools used the kiosk’s rental income to complement the funding of the government-subsidised school meal programme, the perception of reduced profitability could result in compromised motivation for both the kiosk concessionaire and the principal to implement the Guidelines.’[9] |
| **21** | Synthesis output | We identified barriers and facilitators to implementation linked to the following CFIR constructs/sub-constructs: design quality and packaging, cost (intervention characteristics); target group needs and resources, cosmopolitanism, external policy and incentives (outer setting); structural characteristics, readiness for implementation (inner setting); knowledge and beliefs (characteristics of individuals) and engaging, executing (process). The outer setting is the only domain where most of the constructs (three out of four) were present across at least 12 papers. |

References

1. Tong A, Flemming K, McInnes E, Oliver S, Craig J. Enhancing transparency in reporting the synthesis of qualitative research: ENTREQ. BMC Med Res Methodol. 2012;12:181. doi:10.1186/1471-2288-12-181.

2. Malterud K. Qualitative metasynthesis: A research method for medicine and health sciences. Abingdon, Oxon, New York, NY: Routledge; 2019.

3. Thomas J, Harden A. Methods for the thematic synthesis of qualitative research in systematic reviews. BMC Med Res Methodol. 2008;8:45. doi:10.1186/1471-2288-8-45.

4. Consolidated Framework for Implementation Research (CFIR) guide. https://cfirguide.org/.

5. Damschroder LJ, Aron DC, Keith RE, Kirsh SR, Alexander JA, Lowery JC. Fostering implementation of health services research findings into practice: a consolidated framework for advancing implementation science. Implement Sci. 2009;4:50. doi:10.1186/1748-5908-4-50.

6. Gale NK, Heath G, Cameron E, Rashid S, Redwood S. Using the framework method for the analysis of qualitative data in multi-disciplinary health research. BMC Med Res Methodol. 2013;13:117. doi:10.1186/1471-2288-13-117.

7. Critical Appraisal Skills. CASP Qualitative Checklist.

8. Malterud K. Qualitative research: standards, challenges, and guidelines. The Lancet. 2001;358:483–8. doi:10.1016/S0140-6736(01)05627-6.

9. Jensen ML, Gonzalez W, Bolaños-Palmieri C, Monge-Rojas R, Frongillo EA. Implementation of a regulatory food policy to reduce availability of energy-dense foods in Costa Rican high schools. Public Health Nutr. 2021;24:6499–511. doi:10.1017/S1368980021003013.
